# Cathepsin H drives hypoxia-associated inflammatory and angiogenic programs in diabetic retinopathy and represents a potential therapeutic target

**DOI:** 10.7150/ijbs.134125

**Published:** 2026-04-23

**Authors:** Xuehao Cui, Qiuchen Zhao, Jingwen Hui, Yuejun Zhou, Yawen Gong, Wei Zhang, Bidesh Mahata, Patrick Yu-Wai-Man, Quanhong Han

**Affiliations:** 1 Department of Clinical Neurosciences, University of Cambridge, Cambridge CB2 0XY, UK.; 2 John van Geest Centre for Brain Repair, University of Cambridge, Cambridge CB2 0PY, UK.; 3 Cambridge Eye Unit, Addenbrooke's Hospital, Cambridge University Hospitals NHS Foundation Trust, Cambridge CB2 0QQ, UK.; 4 Department of Pathology, University of Cambridge, Cambridge, CB2 1QP, UK.; 5 Cancer Research UK Cambridge Centre and Department of Oncology, University of Cambridge, Cambridge, CB2 0XZ, UK.; 6 Tianjin Eye Hospital, No.4 Gansu Road, Heping District, Tianjin 300020, China.; 7 Tianjin Key Lab of Ophthalmology and Visual Science, Tianjin, China.; 8 School of Medicine, Nankai University, Tianjin, China.

**Keywords:** CTSH, diabetic retinopathy, Mendelian randomization, single cell RNA sequencing, myeloid activation, VEGF, druggable target

## Abstract

Diabetic retinopathy (DR) arises from intertwined inflammatory, metabolic, and hypoxia-driven angiogenic programs, yet upstream regulators coordinating these processes remain incompletely defined. Here, we used an integrative multi-omics and experimental framework to identify cathepsin H (CTSH) as a candidate causal driver of proliferative DR (PDR). By combining GWAS, eQTL, pQTL, and mQTL datasets with Mendelian randomization, summary-data-based Mendelian randomization, and Bayesian colocalization, CTSH emerged as the strongest genetically supported candidate across discovery and validation analyses. In the UK Biobank (UKB), circulating CTSH was elevated in diabetic retinopathy and independently predicted incident disease. Single-cell transcriptomic analyses localized CTSH predominantly to myeloid compartments within fibrovascular membranes and linked CTSH-high states to inflammatory, hypoxic, and angiogenic programs. In high-glucose-stimulated THP-1 monocytes, CTSH promoted reactive oxygen species accumulation, NF-κB activation, and increased IL-6, TNF-α, HIF-1α, and VEGF expression, whereas CTSH silencing reversed these effects. Structure-guided virtual screening identified Eriocitrin as a lead CTSH-binding candidate. In db/db mice, intravitreal Eriocitrin improved inner-retinal function, restored OCTA-derived vascular metrics, and partially rescued retinal structure, with efficacy comparable to anti-VEGF treatment across several endpoints. Molecular analyses further showed coordinated suppression of inflammatory, hypoxic, angiogenic, and NF-κB signaling. Together, these findings identify CTSH as an upstream immunometabolic regulator of DR-related inflammatory and angiogenic biology, with the strongest genetic support observed for PDR, and support CTSH targeting as a potential multi-pathway therapeutic strategy beyond VEGF inhibition.

## 1. Introduction

Diabetic retinopathy (DR) is a leading cause of irreversible vision loss in working-age adults worldwide and reflects chronic microvascular injury induced by prolonged hyperglycemia 1, 2. Proliferative diabetic retinopathy (PDR), the most vision-threatening stage of DR, is characterized by pathological neovascularization and fibrovascular membrane formation at the vitreoretinal interface 3, 4. Advances in metabolic control, panretinal photocoagulation, and intravitreal anti-VEGF therapy have transformed the management of DR 5-7. Many patients exhibit incomplete or transient responses, relapse rapidly, or remain refractory to VEGF blockade. These limitations underscore an urgent need to identify upstream molecular regulators that integrate inflammatory, metabolic, and hypoxic signaling beyond VEGF alone 8.

The pathogenesis of PDR results from the convergence of metabolic dysregulation, innate immune activation, and hypoxia-driven angiogenesis within the diabetic retina 9-12. Chronic hyperglycemia perturbs mitochondrial function and redox homeostasis, generating reactive oxygen species and promoting capillary nonperfusion 13. Local ischemia stabilizes hypoxia-inducible transcription factors (HIFs) and triggers pro-angiogenic programs that promote endothelial proliferation, extracellular matrix remodeling, and formation of fragile neovessels 14-17. In parallel, monocytes, macrophages, microglia, and dendritic cells become activated and infiltrate the retina, releasing IL-1β, IL-6, TNF-α, chemokines, and proteases that amplify vascular leakage and endothelial dysfunction 18-23. Metabolic rewiring, including a glycolytic shift, lipid remodeling, and accumulation of hypoxia-linked metabolites, further reinforces inflammatory activation and HIF-dependent angiogenesis 24-29. Collectively, these interconnected pathways create a self-propagating cycle in which metabolic stress, inflammation, and angiogenesis perpetuate retinal injury 30, 31.

Recent genetic studies have begun to uncover risk loci for DR, but many associated variants reside in noncoding regions or map to genes of uncertain retinal relevance 32, 33. Integrative multi-omics approaches, including expression, protein, and metabolite quantitative trait loci (QTLs) and Mendelian randomization (MR), now enable the identification of molecular drivers with putative causal roles 34, 35. Within this framework, lysosomal proteases have emerged as potential regulators linking immunity, metabolism, and extracellular remodeling 36. Cathepsin H (CTSH), a lysosomal aminopeptidase with immunometabolic and proteolytic functions, is particularly compelling 37. CTSH participates in antigen processing, extracellular matrix degradation, cytokine regulation, and inflammatory activation 38, and recent evidence implicates cathepsin family members, including CTSH, in diabetic inflammation, immune dysregulation, and angiogenesis 39. Given these features, CTSH represents a plausible upstream node that may integrate metabolic stress, innate immune activation, and HIF/VEGF-driven angiogenesis in PDR. However, its causal relevance, cellular specificity, and mechanistic contributions to retinal pathology remain unknown.

In this study, we applied a multi-stage, multi-omics strategy to identify and validate causal regulators of advanced DR, with genetic discovery focused on PDR. We further leveraged UK Biobank (UKB) proteomics and metabolomics, multiple scRNA-seq datasets, diabetic mouse models, human ocular tissues, and *in vitro* perturbation assays to elucidate the molecular, cellular, and functional roles of CTSH across DR-related inflammatory, hypoxic, and angiogenic processes. We further leveraged UKB proteomics and metabolomics, multiple scRNA-seq datasets, diabetic mouse models, human ocular tissues and *in vitro* perturbation assays to elucidate the molecular, cellular and functional roles of CTSH in PDR pathogenesis. Finally, structure-based virtual screening and molecular modeling were used to assess CTSH druggability and nominate Eriocitrin as a lead CTSH-targeting candidate with predicted binding to CTSH, which we evaluated *in vivo* by intravitreal administration in db/db mice benchmarked against anti-VEGF therapy using longitudinal ERG/OCTA/OCT endpoints and terminal retinal molecular profiling. Together, these findings position CTSH as a mechanistic biomarker and a promising therapeutic target that bridges metabolic, inflammatory and angiogenic pathways in PDR.

## 2. Methods and Materials

### 2.1 Study design

We implemented a multi-stage research framework integrating genetic causality, molecular profiling, cellular mapping, longitudinal population analysis, and mechanistic experimentation to establish CTSH as a putative regulatory and therapeutic node in diabetic retinopathy, with specific genetic prioritization focused on PDR. Discovery analyses were performed using summary-data Mendelian randomization (SMR) integrating eQTLGen and GTEx with PDR GWAS datasets from FinnGen and eight European cohorts. Validation analyses consisted of two-sample MR using independent proteomic datasets (INTERVAL, ARIC, AGES) and Bayesian colocalization to infer shared causal variants. Clinical relevance was assessed using longitudinal proteomic and metabolomic profiling from the UKB. Cellular specificity was evaluated by integrating single-cell RNA sequencing (scRNA-seq) with cell-type-specific MR (OneK1K, DICE). Functional mechanisms were examined in diabetic mouse models, human ocular tissues, and high-glucose-stimulated monocyte-lineage cells. Finally, structure-based virtual screening and molecular dynamics (MD) simulations were used to evaluate CTSH druggability and identify candidate inhibitors.

### 2.2 GWAS datasets

We incorporated genetic and multi-omics resources from several large-scale studies. The primary PDR dataset was FinnGen R9 (2,025 cases; 284,826 controls) 40. Replication was performed using a meta-analysis of eight European cohorts (398 PDR cases; 2,848 controls) 41. The DGIdb database served as a reference for identifying druggable genes and known drug-gene interactions 42. Proteomic quantitative trait locus (pQTL) data were obtained from three independent cohorts: AGES-Reykjavik Study (5,764 older adults, ≥67 years, Iceland) 43, ARIC Study (15,792 middle-aged adults from four U.S. communities) 44, and the INTERVAL Study (50,000 UK blood donors, with 3,301 participants profiled for plasma proteomics) 45. Metabolomic quantitative trait locus (mQTL) data were derived from the Canadian Longitudinal Study on Aging (CLSA), which analyzed 1,091 circulating metabolites in 8,299 European participants 46, and the German Chronic Kidney Disease (GCKD) Study (5,217 participants with comprehensive urinary and plasma metabolomics profiling) 47. Cell-type-specific expression quantitative trait locus (eQTL) data were integrated from the DICE Project, which provides cis-eQTLs across human immune cell subtypes 48, and the OneK1K Single-Cell Cohort, which mapped eQTLs in 982 donors and over 1.27 million peripheral blood mononuclear cells 49.

### 2.3 Causal inference and functional annotation approaches

#### 2.3.1 Summary Data-Based MR (SMR)

SMR was used to integrate PDR GWAS summary statistics with eQTL data, applying an FDR-corrected pSMR < 0.05 and HEIDI p > 0.05 as criteria for putative causal associations 50.

#### 2.3.2 Two-Sample Mendelian Randomization

wo-sample MR was conducted using independent SNPs strongly associated with the exposure (p < 5 × 10^-8) as instrumental variables 51. Variants were clumped for linkage disequilibrium using an r² threshold of < 0.01 within a 10,000-kb window. For each analysis, we recorded the number of retained instruments and calculated instrument strength using the F statistic; instruments with F > 10 were considered sufficiently strong to reduce the likelihood of weak-instrument bias. When only a single valid instrument was available, the Wald ratio was applied; otherwise, the primary method was inverse-variance weighted (IVW), with MR-Egger and weighted median used as sensitivity analyses when instrument number permitted. Horizontal pleiotropy was evaluated using the MR-Egger intercept and MR-PRESSO, and heterogeneity was assessed using Cochran's Q test 52. Analyses were implemented in R with the TwoSampleMR package. Detailed instrument numbers and corresponding statistics for each analysis are provided in the Supplementary Tables. Significance was defined as p < 0.05.

#### 2.3.3 Colocalization analysis

Colocalization analysis was performed using the coloc package to estimate posterior probabilities for five hypotheses (PP0-PP4), with PP4 representing the probability that the exposure and outcome share a common causal variant. Strong evidence of colocalization was defined as PP4 > 0.8, and moderate evidence as PP4 > 0.5 53. Regional colocalization was interpreted jointly with MR effect estimates and instrument characteristics to reduce overinterpretation of isolated associations.

#### 2.3.4 Protein-Protein Interaction (PPI) network analysis

Candidate proteins from MR analyses were mapped to STRING, retaining high-confidence interactions (score > 0.7). Networks were visualized using Cytoscape, and modules and hub proteins were identified 54.

#### 2.3.5 Pathway Enrichment Analysis

Kyoto Encyclopedia of Genes and Genomes (KEGG) and Gene Ontology (GO) enrichment analyses were performed using the clusterProfiler R package 55. Overrepresentation was tested with a hypergeometric distribution and FDR-adjusted results. Pathways with adjusted p < 0.05 were considered significantly enriched.

#### 2.3.6 Mediation MR

Mediation MR was conducted in a two-step framework. The first step estimated the causal effect of exposure on the mediator, and the second assessed the mediator's impact on the outcome. Indirect effects were calculated using the product of coefficients, with standard errors derived by the delta method. Significance was defined as p < 0.05 56.

#### 2.3.7 Multi-omics framework for causal gene identification in PDR

Based on these multi-layered analyses, we established a tiered classification system in which genes meeting all 9 criteria were defined as Tier 1, those meeting more than four criteria as Tier 2, and those meeting three criteria as Tier 3. This multi-stage strategy, combining SMR, MR, colocalization, and tiered prioritization, enabled a systematic evaluation of causal genes for PDR at multiple molecular levels.

#### 2.3.8 Mediation framework for CTSH-metabolite-PDR axis

To explore metabolic pathways linking CTSH to PDR, we performed MR analyses using CTSH QTLs against the CLSA metabolite GWAS to identify metabolites associated with CTSH. Metabolites significantly associated with CTSH were subjected to KEGG pathway enrichment analysis, with increased and decreased metabolites analyzed separately using MetaboAnalyst and clusterProfiler. Subsequently, mediation MR integrating CTSH-metabolite and metabolite-PDR associations was performed to estimate indirect effects and mediation proportions, providing insight into potential metabolic pathways through which CTSH may influence PDR.

### 2.4 Validation of CTSH expression in mouse models and human ocular tissues

#### 2.4.1 Mouse Model and Tissue Collection

A streptozotocin (STZ)-induced diabetic retinopathy model was used. Diabetic status was confirmed by fasting glucose and clinical evaluation. Mice were euthanized at predefined time points; peripheral blood was collected for leukocyte RNA, and retinas were harvested for RNA and protein analyses. All animal procedures followed IACUC-approved protocols.

#### 2.4.2 Human Ocular Tissue Collection and Processing

With IRB approval and informed consent, ocular tissues were collected intraoperatively from three groups: PDR fibrovascular membranes (FMs), diabetic non-DR epiretinal/FMs, and non-diabetic controls (n=3 per group). Samples were snap-frozen and stored at -80°C. CTSH protein expression was assessed by Western blot, in parallel with mouse samples. All analyses were blinded to group assignment.

#### 2.4.3 Molecular Assays: qPCR and Western Blot

qPCR was performed on RNA from mouse leukocytes and retinas; Western blotting was conducted on mouse and human retinal membranes. Total RNA was extracted with TRIzol, converted to cDNA, and analyzed by real-time PCR using GAPDH or β-actin as reference genes. Protein was extracted with RIPA buffer, quantified by BCA, resolved by SDS-PAGE, and transferred to PVDF membranes. Membranes were probed with anti-CTSH and appropriate loading controls, followed by HRP-secondary antibodies and ECL detection. Densitometry was performed using ImageJ.

#### 2.4.4 Quality Control and Statistical Analysis

All samples were processed blindly. Batch-to-batch consistency was monitored using internal controls. Statistical comparisons were based on normality tests, with t-tests or nonparametric methods used as appropriate. One-way ANOVA or rank-based tests with post hoc correction were applied for multi-group comparisons. Significance was defined as p < 0.05; multiple testing was adjusted using BH-FDR.

### 2.5 ScRNA-seq data processing and Sc-MR analyses

ScRNA-seq datasets derived from fibrovascular membrane (FM) and blood samples from DR patients were retrieved from the Gene Expression Omnibus under accession numbers GSE165784, GSE248284, and GSE245561 57-59. Cells with fewer than 200 or more than 7,500 detected genes, or with > 20% mitochondrial gene expression, were excluded. The filtered count matrices were normalized using the SCTransform algorithm in Seurat (version 5.0.3) 60. Highly variable genes (n = 2,000) were selected for dimensionality reduction using principal component analysis (PCA), and the number of principal components used for downstream analyses (n = 15) was determined based on ElbowPlot inspection. To account for batch effects across datasets and donors, data integration was performed using the Harmony algorithm (v1.2.0). Integration quality was evaluated using multiple complementary metrics. Batch mixing and integration performance were assessed using the k-nearest neighbor batch effect test (kBET) and the Local Inverse Simpson's Index (LISI). kBET rejection rates were calculated to quantify batch mixing across integrated datasets, while integration LISI (iLISI) scores were used to evaluate donor mixing across cell populations. Donor contribution to each cell cluster was additionally examined to ensure that no individual donor dominated cluster composition after integration.

Unsupervised clustering was carried out by constructing a Shared Nearest Neighbor (SNN) graph using Seurat's FindNeighbors function, followed by clustering with the FindClusters function at a resolution of 0.6. Non-linear dimensionality reduction was conducted using Uniform Manifold Approximation and Projection (UMAP) or t-distributed Stochastic Neighbor Embedding (t-SNE), via Seurat's RunUMAP and RunTSNE functions, respectively. Cell types were annotated based on differentially expressed genes (DEGs) identified using the FindAllMarkers function. Marker genes and DEGs were used to define canonical immune subsets and to explore transcriptional differences. Pathway enrichment analyses were conducted using the clusterProfiler package (v4.6.2) 61 to perform GSEA 62.

To investigate cell-type-specific genetic effects of CTSH, we integrated cell-type eQTL data from the DICE project and the OneK1K single-cell eQTL cohort with the FinnGen R9 PDR GWAS in a two-sample Mendelian randomization framework. Independent cis-eQTL variants associated with CTSH expression were used as instrumental variables after LD clumping (r² < 0.01). The number of instruments varied across cell types depending on available eQTL signals. Effect estimates were scaled per genetically predicted standard deviation increase in CTSH expression. The primary MR method was inverse-variance weighted (IVW), and sensitivity analyses were conducted where instrument numbers permitted.

### 2.6 Proteomic and metabolomic analyses in the UKB cohort

#### 2.6.1 Cohort and data curation

This analysis utilized the prospective UKB cohort 63. DR diagnoses were derived from linked hospital records, screening data, and ophthalmic procedure codes. Data curation included removing consent withdrawals, excluding samples with missing key covariates, filtering technical outliers (> 5 SD), and imputing values below the limit of detection. Continuous variables were log- or z-transformed. Time-to-event data were generated with censoring at death, loss to follow-up, or administrative end. For proteomic analyses, the platform-normalized protein abundance values provided by UK Biobank were used as the starting input. CTSH levels were standardized to z-scores for regression and survival analyses to facilitate effect-size comparability across models. Continuous covariates with skewed distributions were log-transformed where appropriate before standardization. Values below the limit of detection were imputed according to the assay-specific preprocessing pipeline, and samples with extreme technical values (> 5 SD from the mean) were excluded as outliers.

#### 2.6.2 Longitudinal risk modeling

CTSH (modeled both continuously and by quantiles) was entered as the exposure in Cox proportional hazards models for time to incident DR. Sequential models were specified to improve transparency. The core model adjusted for age, sex, ethnicity, socioeconomic deprivation, smoking status, alcohol intake, body mass index, systolic blood pressure, antihypertensive medication use, lipid-lowering medication use, diabetes status, and relevant technical covariates related to proteomic measurement. The extended model additionally incorporated available metabolic traits, including HbA1c, where available in the corresponding analytic subset. In diabetes-restricted analyses, we further adjusted for baseline glycemic status and major cardiometabolic covariates, but diabetes duration and insulin therapy were not consistently available for all included participants and therefore were not uniformly incorporated into the primary models; this was considered in the interpretation of results. Proportional hazards were checked using Schoenfeld residuals. Kaplan-Meier curves with log-rank tests were generated, and time-dependent ROC-AUCs at 3, 5, and 10 years together with Harrell's C-index were used to quantify model discrimination. Missing covariates were handled using multiple imputation by chained equations (MICE). Twenty imputed datasets were generated, and model estimates were combined using Rubin's rules. The imputation model included the exposure, outcome indicator, follow-up time, and all covariates used in the fully adjusted analyses.

#### 2.6.3 Protein correlation and functional stratification

Spearman correlations between CTSH and the complete proteome were computed; proteins were functionally annotated using GO/Reactome and categorized into angiogenesis, immune, and inflammation classes. Correlation networks were constructed with FDR control for multiple testing; partial correlations were estimated in multivariable frameworks to account for confounding.

#### 2.6.4 Proteome-metabolome integration

In participants with both layers, multi-block integration (CCA/PLS-DA/sparse multi-block PLS, such as sGCCA) characterized the proteome-metabolome coupling; multivariable regressions tested independent associations between CTSH and key metabolic pathways; network mapping and pathway enrichment analyses contextualized system-level features.

#### 2.6.5 Statistical methods and software

Continuous variables were log- or rank-transformed as appropriate; group contrasts used linear/GLM models; associations used Spearman/partial correlations; survival analyses used Cox models and time-dependent ROC. Diagnostics included residual checks, collinearity (VIF), nonlinearity (splines), and influence measures. Multiple testing was controlled using the BH-FDR. Analyses were implemented in R (survival, survminer, timeROC/pROC, rms, glmnet, limma, sva, mixOmics, igraph, data wrangling/visualization). Unless otherwise specified, two-sided p < 0.05 defined statistical significance; effect sizes were standardized per SD.

### 2.7* In-Vitro* Functional Assays under High Glucose with CTSH Perturbation

Human THP-1 monocytes (ATCC TIB-202) were maintained in RPMI-1640 medium (Gibco) supplemented with 10% fetal bovine serum (FBS), penicillin (100 U/mL), and streptomycin (100 µg/mL) at 37 °C in a humidified 5% CO₂ incubator. Cells were divided into four experimental groups: (1) standard control (NC, 5.5 mM glucose), (2) high glucose (HG, 25 mM), (3) HG + CTSH overexpression (HG + oe-CTSH), and (4) HG + CTSH knockdown (HG + si-CTSH). CTSH overexpression was induced by transfection with a plasmid pcDNA3.1-CTSH expression plasmid, and CTSH knockdown was achieved using specific small interfering RNAs (siRNAs) with scrambled siRNA as a negative control. Transfections were performed using Lipofectamine 3000 (Thermo Fisher) according to the manufacturer's protocol. Transfection efficiency was confirmed by both qPCR and Western blotting. Each group included six biological replicates, and all experiments were independently repeated at least twice (Table S34).

#### 2.7.1 Measurement of Reactive Oxygen Species and Gene Expression

Intracellular reactive oxygen species (ROS) levels were measured using the DCFH-DA fluorescent probe (Beyotime S0033S). Cells were incubated with 10 µM DCFH-DA for 30 min at 37 °C in the dark, washed three times with PBS, and analyzed immediately using a fluorescence microplate reader (excitation = 488 nm, emission = 525 nm). Fluorescence intensity was normalized to viable cell counts. For gene expression analyses, total RNA was extracted using TRIzol reagent (Invitrogen) and reverse-transcribed to cDNA with the PrimeScript RT kit (Takara). Quantitative PCR was performed using SYBR Green Premix Ex Taq II (Takara) on a LightCycler 480 (Roche). Expression levels of CTSH and downstream target genes—including VEGF, IL-6, TNF-α, and HIF-1α—were quantified. GAPDH or β-actin served as an internal reference. Relative gene expression was calculated using the 2⁻ΔΔCt method.

#### 2.7.2 Protein Expression and Phosphorylation Analysis

Cells were lysed in RIPA lysis buffer (Beyotime, P0013C) containing protease inhibitor cocktail (Beyotime, P1005), and lysates were clarified by centrifugation at 12,000 rpm for 15 min at 4 °C. Protein concentrations were measured using a BCA protein assay kit (MDL, MD913053). Equal amounts of protein were separated by SDS-PAGE and transferred onto nitrocellulose membranes (0.22 μm, GVS, ISEQ00010) at 100 V for 1 h.

Membranes were blocked with 3% non-fat milk in TBST for 1 h and incubated overnight at 4 °C with primary antibodies against CTSH (Santa Cruz, sc-398527, 1:1000), VEGF (Proteintech, 19003-1-AP, 1:2000), IL-6 (Proteintech, 21865-1-AP, 1:1000), TNF-α (Proteintech, 26405-1-AP, 1:1000), HIF-1α (Proteintech, 20960-1-AP, 1:2000), NF-κB p65 (Proteintech, 10745-1-AP, 1:2000), phosphorylated NF-κB p65 (Proteintech, 80379-2-RR, 1:2000), IκBα (Proteintech, 10268-1-AP, 1:5000), phosphorylated IκBα (Proteintech, 82349-1-RR, 1:1000), Caspase-3 (Proteintech, 68773-1-Ig, 1:5000), and β-actin (MDL, MD6553, 1:1000). After washing with TBST, membranes were incubated with HRP-conjugated secondary antibodies (Servicebio, C030205 or C030212) for 1 h at room temperature. Protein signals were detected using enhanced chemiluminescence and imaged with a ChemiScope 6100 system.

#### 2.7.3 Immunofluorescence and Co-Localization Analysis

For immunofluorescence staining, THP-1 cells were seeded on sterile glass coverslips, fixed with 4% paraformaldehyde for 15 min, permeabilized with 0.2% Triton X-100 for 10 min, and blocked with 5% BSA for one h. Cells were incubated overnight at 4 °C with primary antibodies against CTSH (1:200) and either VEGF (1:200) or IL-6 (1:200). After washing, Alexa Fluor 488- and 594-conjugated secondary antibodies (Invitrogen A-11008, A-11012, 1:500) were applied for one h at room temperature. Nuclei were counterstained with DAPI (1 µg/mL). Images were acquired on a Leica SP8 confocal microscope using a 63× oil-immersion objective under identical exposure parameters.

Fluorescence intensity and co-localization were analyzed using ImageJ with the Coloc2 plugin. Pearson's correlation and Manders' coefficients were calculated for each field. At least five random fields per biological replicate and ≥100 cells per group were quantified. Data is presented as mean ± SEM, with individual points representing independent biological replicates.

#### 2.7.4 Co-Immunoprecipitation (Co-IP)

Protein-protein interactions were evaluated by co-immunoprecipitation. Whole-cell lysates (500 µg protein) were incubated with two µg anti-CTSH antibody (Abcam ab133650) or IgG control overnight at 4 °C with gentle rotation, followed by incubation with 30 µL Protein A/G magnetic beads (Thermo Fisher 88802) for two hours. The beads were washed five times in ice-cold RIPA buffer containing 0.1% NP-40 and eluted with SDS sample buffer. Input, IgG, and immunoprecipitated fractions were analyzed by Western blot for VEGF, IL-6, and HIF-1α to confirm specific CTSH-associated complexes.

#### 2.7.5 Statistical Analysis

All quantitative results were summarized at the biological replicate level. Normality was assessed using the Shapiro-Wilk test. Group differences were analyzed using one-way ANOVA followed by Tukey's post hoc test for normally distributed data or Kruskal-Wallis test for non-parametric data. Two-sided p < 0.05 was considered statistically significant, and multiple comparisons were adjusted using the Benjamini-Hochberg false discovery rate (FDR) method. Statistical analyses were performed using GraphPad Prism 10 and R (v4.3.2).

### 2.8 Molecular docking and molecular dynamics simulation

#### 2.8.1 Molecular docking

A total of 24,123 compounds from the TargetMol and MCE libraries were prepared using LigPrep with the OPLS3e force field. The crystal structure of CTSH (PDB ID: 6CZK) was retrieved from the RCSB Protein Data Bank and processed using the Protein Preparation Wizard in Schrödinger. The binding pocket was defined within a 15 × 15 × 15 Å grid box. Virtual screening was conducted in three sequential stages using Glide, including standard precision (SP) docking, extra precision (XP) docking, and MM-GBSA rescoring. Final candidates were selected based on docking scores, binding conformations, and structural inspection.

#### 2.8.2 Molecular dynamics simulation

Top ligand-protein complexes were subjected to 100 ns molecular dynamics simulations in Schrödinger with the OPLS3e force field. System stability was assessed by root mean square deviation (RMSD), root mean square fluctuation (RMSF), radius of gyration (Rg), solvent-accessible surface area (SASA), and hydrogen bond monitoring. Binding free energy (ΔG_binding) was estimated using MM-GBSA, and van der Waals and electrostatic contributions were analyzed to characterize ligand-protein interactions.

#### 2.8.3 Functional evaluation of MD-selected CTSH-binding candidates in monocyte-lineage cells

To evaluate the biological effects of top MD-identified CTSH-targeting candidate compounds, *in vitro* assays were conducted using THP-1 monocytes under high-glucose conditions. Three compounds—Eriocitrin, Tenuifoliside B, and AP-III-a4—were selected based on their stable binding profiles in 100-ns molecular dynamics simulations. THP-1 cells were treated with normal glucose (5.5 mM), high glucose (25 mM), or high glucose plus compound for 24 hours. Total protein was extracted, quantified by BCA assay, and analyzed by Western blot. Key inflammatory and angiogenic markers—VEGF, IL-6, TNF-α, HIF-1α, total and phosphorylated NF-κB p65/IκBα, and caspase-3—were detected, with β-actin as loading control. Chemiluminescent detection and densitometric analysis were performed under standardized conditions.

### 2.9 *In vivo* evaluation of CTSH targeting in db/db diabetic retinopathy mice

#### 2.9.1 Mice and study design

Spontaneous type 2 diabetic BKS-db/db mice were used as an *in vivo* model of diabetic retinal injury, with age-matched syngeneic normal BKS mice serving as non-diabetic controls (NC). Mice were maintained under specific pathogen-free (SPF) conditions and standard chow. Animals entered the study at 11 weeks of age and were maintained to 12 weeks of age, at which point treatment was initiated. At this age, the db/db model primarily reflects early-to-intermediate diabetic retinal abnormalities, including microvascular dysfunction, inflammatory activation, and neural impairment, rather than fully developed proliferative diabetic retinopathy. Four experimental groups were defined: NC (normal control), DR (disease model), Anti-VEGF (Aflibercept), and CTSH targeting (Eriocitrin). At 12 weeks of age, mice in the Anti-VEGF and Eriocitrin groups received a single unilateral intravitreal injection (one eye per mouse). Animals were anesthetized with 2% isoflurane, pupils were dilated using compound tropicamide, and 1 μL of solution was injected into the vitreous cavity using a microsyringe. Doses were: aflibercept 2.0 μg/eye (Anti-VEGF group) and Eriocitrin 0.5 μg/eye. All animal procedures were approved by the institutional committee under protocol (KY-2023WJW003) and were performed in accordance with relevant guidelines (ARVO Statement for the Use of Animals in Ophthalmic and Vision Research).

#### 2.9.2 Retinal ERG, OCT and OCTA acquisition and quantitative metrics

Functional and vascular phenotyping was performed before dosing (week 0, baseline) and after treatment (weeks 3 and 6 post injection), including electroretinography (ERG), optical coherence tomography (OCT), and OCT angiography (OCTA). Retinal function was assessed by ERG at the indicated timepoints, with readouts including dark-adapted b-wave amplitudes (3.0 and 10.0), oscillatory potential OS1 amplitude, and 30-Hz flicker amplitude. Retinal structure and microvasculature were assessed using OCT and OCTA at the indicated timepoints. OCT structural metrics included inner retinal layer (IRL) thickness and RPE-choroid complex thickness. OCTA metrics included vessel density (VD), microvascular density (MVD), vessel skeleton density (VSD), vessel tortuosity (VTor), and vessel total length (VT). Quantification was performed consistently across groups and timepoints using the same analysis workflow.

#### 2.9.3 Retinal tissue collection, RT-qPCR and Western blotting

Following completion of imaging and ERG measurements, mice were euthanized and retinas were carefully dissected for molecular analyses. For RNA analysis, total RNA was extracted using TRIzol reagent (Aidlab, RN0102), and cDNA was synthesized using ExonScript RT SuperMix with dsDNase (ExonGene, A502) following the manufacturer's protocol. Quantitative PCR was performed using SYBR Green Master Mix (ABI Invitrogen, 4472920) with cycling conditions of 95 °C for 5 min followed by 40 cycles of 95 °C for 10 s, 58 °C for 20 s, and 72 °C for 20 s. Actin was used as the internal control, and primer sequences are listed in the Supplementary Table.

For Western blot analysis, retinal tissues were homogenized in RIPA lysis buffer (Beyotime, P0013C) supplemented with protease inhibitor cocktail (Beyotime, P1005). Lysates were centrifuged at 12,000 rpm for 15 min at 4 °C and protein concentrations were determined using a BCA protein assay kit (MDL, MD913053). Equal amounts of protein were separated by SDS-PAGE and transferred to nitrocellulose membranes (0.22 μm, GVS, ISEQ00010) at 100 V for 1 h. Membranes were blocked with 3% non-fat milk in TBST for 1 h at room temperature and incubated overnight at 4 °C with primary antibodies against β-actin (MDL, MD6553, 1:1000), CTSH (Santa Cruz, sc-398527, 1:1000), VEGF (Proteintech, 19003-1-AP, 1:2000), TNF-α (Proteintech, 26405-1-AP, 1:1000), IL-6 (Proteintech, 21865-1-AP, 1:1000), HIF-1α (Proteintech, 20960-1-AP, 1:2000), Caspase-3 (Proteintech, 68773-1-Ig, 1:5000), NF-κB p65 (Proteintech, 10745-1-AP, 1:2000), phosphorylated NF-κB p65 (Proteintech, 80379-2-RR, 1:2000), IκBα (Proteintech, 10268-1-AP, 1:5000), and phosphorylated IκBα (Proteintech, 82349-1-RR, 1:1000). After washing with TBST, membranes were incubated with HRP-conjugated secondary antibodies (Servicebio, C030205 or C030212) for 1 h at room temperature. Protein bands were visualized using enhanced chemiluminescence and imaged using a ChemiScope 6100 imaging system.

#### 2.9.4 Retinal histology and vascular leakage

For histological assessment, enucleated eyes were fixed in 4% paraformaldehyde, paraffin-embedded and sectioned at 5 μm. Sections were stained with hematoxylin and eosin (H&E) and imaged to evaluate retinal laminar organization and structural alterations. Retinal vascular permeability was assessed by fluorescein leakage imaging. Following systemic fluorescein administration, fundus fluorescence images were acquired under identical settings across groups. Increased extravascular fluorescence signal was interpreted as vascular leakage.

#### 2.9.5 Flow cytometry analysis of retinal immune cells

Retinal immune cell infiltration was analyzed by flow cytometry. Retinal tissues were dissociated into single-cell suspensions and stained with fluorophore-conjugated antibodies against CD45, CD11b and MHC-II to identify leukocytes, myeloid cells and activated immune cells. Samples were analyzed by flow cytometry using consistent gating strategies across groups.

## 3. Result

### 3.1 Multi-omics discovery prioritizes CTSH as a causal gene for PDR

Guided by criteria mentioned in Methods, including SMR significance after FDR correction with non-significant HEIDI, replication across cohorts, support from two-sample MR, and colocalization evidence, we made an integrated evidence form (Table 1, Figure S1A-F, Table S1-6, S10). Among all candidates, CTSH was the only gene that met all discovery and validation criteria and was identified as the only tier-1 gene. CYP21A2, MICB, CCNE2, and TP53INP1 were classified as tier 2 because one or more validation steps were not met, while PPIP5K2, XRCC1, C4B, C4A, HLA-C, and ZBTB22 were grouped into tier 3 with only partial support. This multi-layer framework, therefore, highlights CTSH as the most robust causal candidate for PDR. Summary of candidate genes prioritized through expression quantitative trait locus (eQTL), summary-based Mendelian randomization (SMR), two-sample MR, and genetic colocalization analyses. Evidence was integrated across multiple European cohorts and omics layers to classify genes into three confidence tiers: Tier 1 (CTSH) - met all discovery and validation criteria; Tier 2 - showed partial validation across datasets; Tier 3 - demonstrated limited or cohort-specific support. PP4 values indicate colocalization probability; PP4 > 0.8 indicates strong evidence, and 0.5 < PP4 ≤ 0.8 indicates moderate evidence of shared causality.

### 3.2 Proteomic MR validates the role of CTSH in PDR and links CTSH to glycemic traits

**Figure 2:**
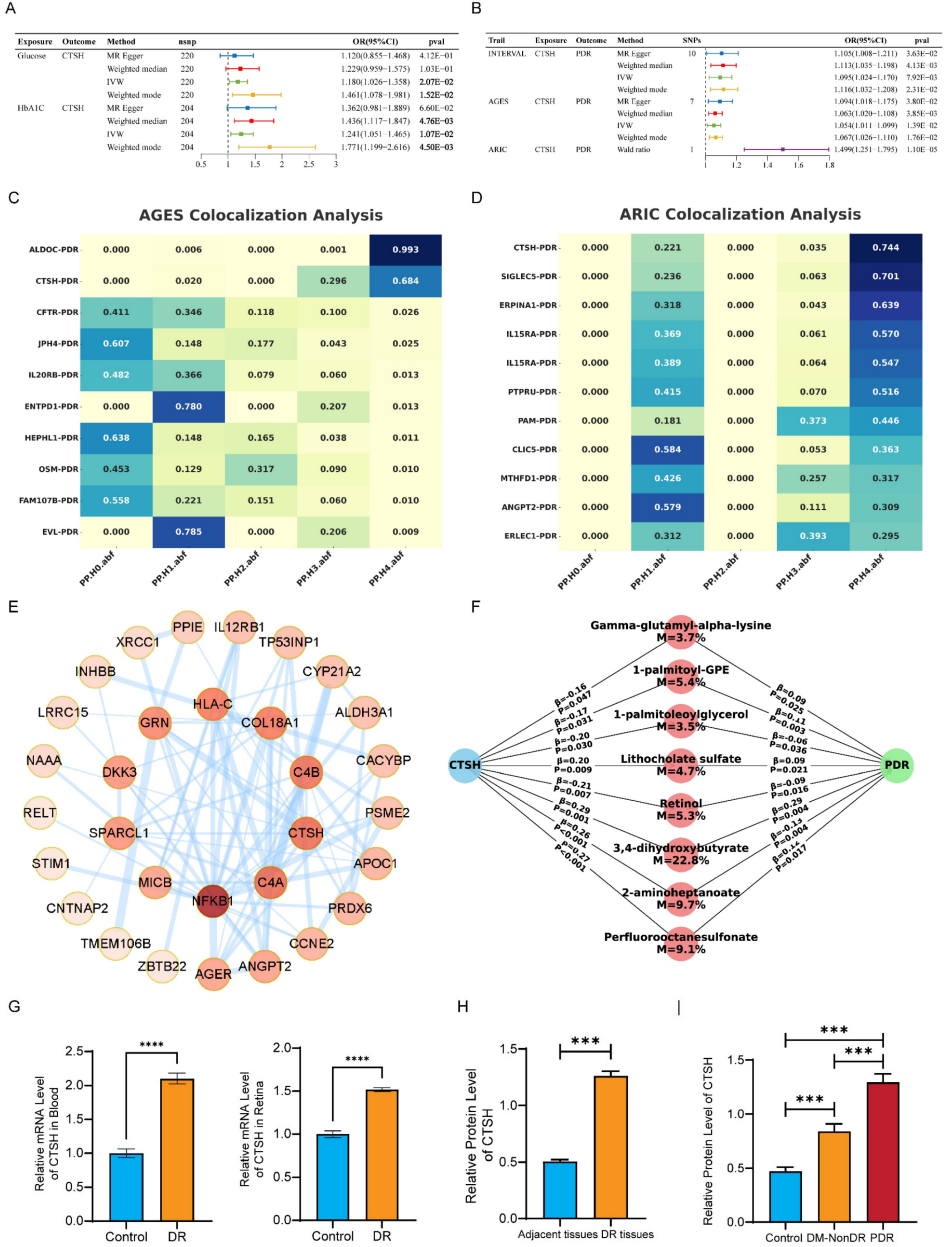
** Genetic and molecular evidence supporting CTSH causality in PDR. (A-B)** Two-sample Mendelian randomization analyses demonstrating association between genetically predicted CTSH and PDR across multiple MR methods. **(C-D)** Bayesian colocalization analyses in AGES and ARIC cohorts indicating shared causal variants between CTSH locus and PDR risk. **(E)** Protein-protein interaction network linking CTSH to angiogenic and inflammatory mediators. **(F)** Mediation analysis identifying metabolites partially mediating the CTSH-PDR relationship. **(G-H)** Validation of CTSH upregulation in human DR tissues at mRNA and protein levels.

Two-sample MR using large-scale proteomic GWAS data supported an association between genetically predicted circulating CTSH levels and PDR (Figures 2B and S3A-B). Higher CTSH levels were associated with increased PDR risk in the INTERVAL cohort (IVW OR = 1.095, 95% CI 1.024-1.170, P = 7.92×10⁻³) and the AGES cohort (OR = 1.054, 95% CI 1.011-1.099, P = 1.39×10⁻²), while a Wald ratio analysis in ARIC showed a larger effect (OR = 1.499, 95% CI 1.251-1.795, P = 1.10×10⁻⁵) (Tables S7-S9). Results from additional MR methods were directionally consistent. MR analyses further indicated positive associations of glucose (IVW OR = 1.180, 95% CI 1.026-1.358, P = 2.07×10⁻²) and HbA1c (IVW OR = 1.241, 95% CI 1.051-1.465, P = 1.07×10⁻²) with CTSH levels (Figure 2A), suggesting that chronic hyperglycemia may contribute to CTSH upregulation. Colocalization analyses provided supportive evidence for a shared genetic signal between the CTSH locus and PDR in AGES (PP4 = 0.684) and ARIC (PP4 = 0.744) (Figure 2C-D; Figure S3C-F; Tables S11-S12). Consistently, PPI and KEGG analyses placed CTSH within immune and inflammatory pathways, including complement signaling, antigen processing and presentation, T/B cell receptor signaling, and Th17 differentiation (Figure 2E; Figure S2A-D). Together, these genetic and pathway-level findings implicate CTSH in immune-inflammatory networks relevant to PDR, providing a mechanistic framework for the functional experiments described below.

### 3.3 Metabolomic MR and mediation suggest a CTSH-metabolite-PDR axis

To further explore metabolic mechanisms linking CTSH to PDR, we conducted metabolomic MR analyses in two independent cohorts. In CLSA, 76 PDR-associated metabolites were identified (42 increased and 34 decreased), whereas 121 significant associations were observed in GCKD (65 increased and 56 decreased) (Tables S26-S27). Pathway enrichment revealed consistent clustering of metabolic pathways across cohorts. In CLSA, upregulated metabolites were enriched in primary bile acid biosynthesis, histidine metabolism, and glutathione metabolism, while downregulated metabolites were associated with branched-chain amino acid degradation and pyrimidine metabolism (Figure S5A-B). GCKD showed similar enrichment patterns involving purine, tryptophan, and unsaturated fatty acid metabolism, as well as carbohydrate and TCA cycle pathways (Figure S5C-D). To evaluate potential mediation, we performed MR analyses of CTSH on 1,091 metabolites in the CLSA dataset. CTSH-associated metabolites were enriched in riboflavin, sphingolipid, and lipid metabolism, while decreased metabolites were linked to pantothenate and CoA biosynthesis, glutathione metabolism, and carbohydrate pathways (Figure S5E-F). Mediation mapping highlighted several candidate metabolites—including perfluorooctanesulfonate, 2-aminoheptanoate, 3,4-dihydroxybutyrate, retinol, lithocholate sulfate, and glycerolipids—with estimated mediation proportions ranging from 3% to 23% (Figure 2F; Table S28). Together, these findings suggest that CTSH may influence PDR not only through inflammatory and angiogenic pathways but also through broader metabolic alterations.

### 3.4* In vivo* mouse model and human tissues confirm CTSH overexpression in DR/PDR

We next validated CTSH expression in animal models and human ocular specimens. In the STZ-induced DR mouse model, quantitative PCR showed that *CTSH* mRNA was significantly elevated in both peripheral blood and retinal tissue compared with controls (Figure 2G). Consistently, Western blot analysis revealed a marked increase in CTSH protein levels in DR mouse retinas relative to adjacent control tissues (Figure 2H).

In human samples, western blotting of ocular membranes showed that CTSH protein abundance was substantially higher in fibrovascular proliferative membranes from PDR patients than in those from diabetic patients without DR (DM-NonDR) or non-diabetic controls (Figure 2I). Notably, CTSH expression showed a stepwise increase from controls to DM-NonDR and was highest in PDR tissues, supporting a disease-specific elevation of CTSH in advanced retinopathy.

### 3.5 Single-cell analyses localize CTSH to myeloid compartments and pro-angiogenic/inflammatory programs

**Figure 3:**
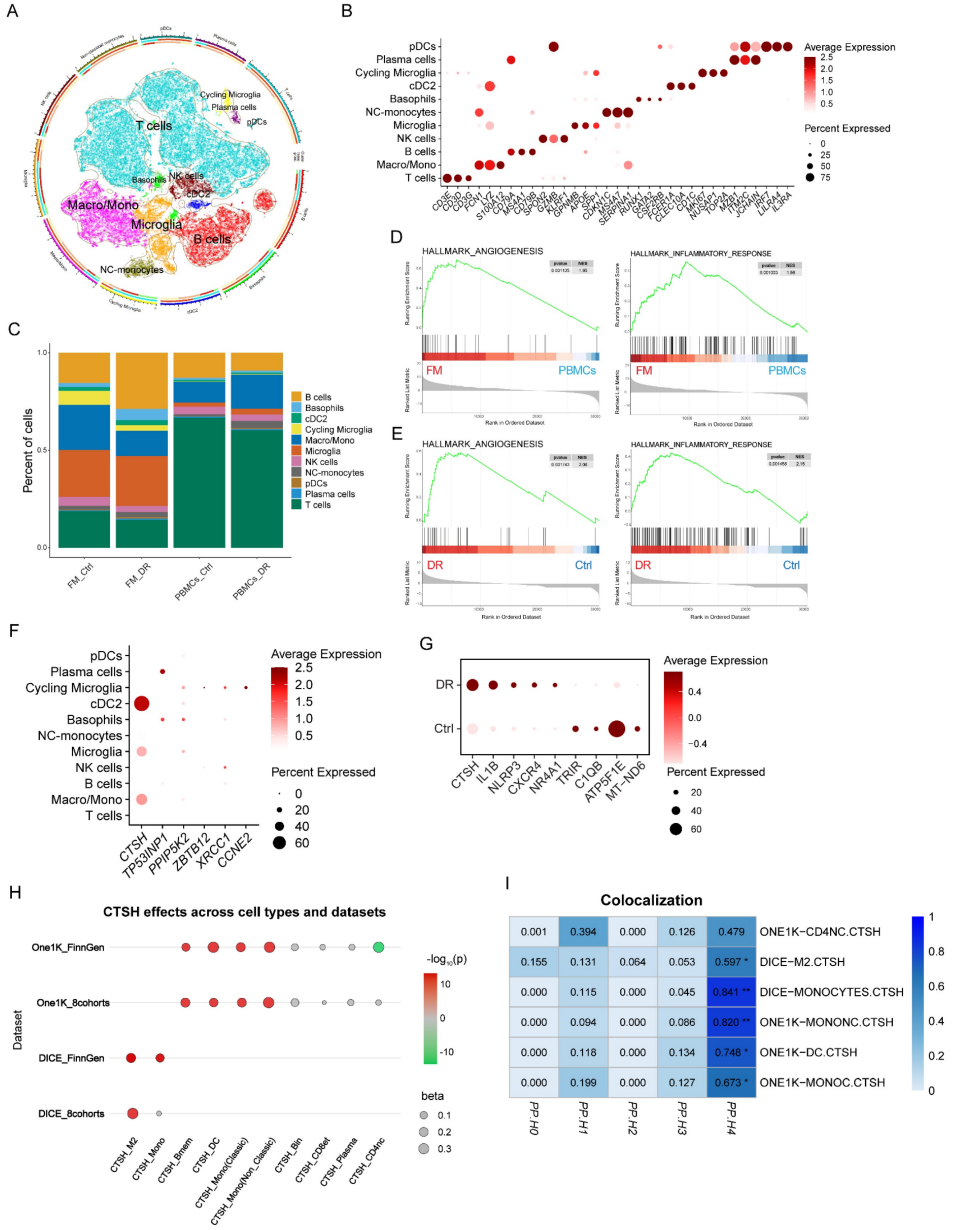
** CTSH levels associate with PDR risk and vascular-inflammatory signaling. (A)** Cox proportional hazards models showing CTSH association with incident DR (unadjusted and covariate-adjusted models). **(B)** Distribution of scaled CTSH expression in control and DR groups. **(C-D)** Dose-response relationship between CTSH levels (z-score) and hazard ratio over time. **(E)** CTSH-centered interaction network linking vascular and inflammatory signaling proteins. **(F)** Spearman correlations between CTSH and inflammatory/metabolic proteins. **(G-H)** Feature loading analyses identifying CTSH-related inflammatory clusters. **(I-K)** Subgroup Cox models and nonlinear risk curves for CTSH across diabetic populations.

In light of previous findings implicating CTSH in the pathogenesis of DR, we further examined its expression across different cell types in DR by integrating scRNA-seq data from fibrous membrane (FM) tissues and peripheral blood mononuclear cells (PBMCs) collected from 12 DR patients and four non-diabetic retinopathy (NDR) controls 1-3. An integrated immune atlas comprising 57,674 cells was constructed to investigate the systemic and local immune alterations in DR (Figure 3A). Major immune lineages, including T cells, B cells, and diverse myeloid subpopulations, were identified based on canonical markers (Figure 3B).

Immune compositions differed markedly between FM and PBMC samples. FM tissues exhibited an increased proportion of myeloid cells and a reduced number of T cells compared to PBMCs. At the same time, DR-specific FM samples showed enrichment of B cells and depletion of macrophages, opposite to the trends observed in matched PBMCs (Figure 3C). Beyond compositional changes, GSEA revealed significantly elevated pathways related to angiogenesis, inflammation, and hypoxia in FM relative to PBMCs, indicating tissue-specific immune activation (Figure 3D, Figure S7A). Notably, these pathways were also more strongly enriched in FM samples from DR patients compared to NDR controls (Figure 3E, Figure S7B). Given that several genes identified in Figure 2, particularly CTSH, were associated with PDR risk and with inflammatory and angiogenic signatures, we next examined their expression across immune cell subsets. *CTSH* was highly expressed in myeloid populations, including monocytes/macrophages, cDC2, and microglia (Figure 3F). We further observed elevated *CTSH* expression in myeloid cells from DR patients compared with non-DR controls, accompanied by upregulation of pro-inflammatory genes such as IL1B and NLRP3 and downregulation of mitochondrial and functional genes, including *ATP5F1E* and *C1QB* (Figure 3G).

To examine whether specific cell types mediate the genetic regulation of CTSH, we conducted two-sample MR by integrating cell-type eQTLs from DICE and OneK1K with PDR GWAS from FinnGen R9 (Table S23-24). Significant positive effects were concentrated in myeloid cell types, particularly monocytes and dendritic cells, while lymphoid lineages showed weak or no effects (Figure 3H). Bayesian colocalization analysis provided strong support for colocalization in monocytes (PP4 = 0.8406) and non-classical monocytes (PP4 = 0.8202), whereas dendritic cells showed only moderate supportive evidence (PP4 = 0.7480) (Table S25). Additional supportive evidence was found in monocyte-like cells with PP4 equal to 0.6734 and M2-like macrophages with PP4 equal to 0.5970, while CD4 naïve cells did not reach the threshold with PP4 equal to 0.4785 (Figure 3I). Together, these findings suggest that CTSH may contribute to PDR primarily through myeloid compartments within fibrovascular membranes, which are embedded in angiogenesis- and inflammation-driven programs.

### 3.6 UKB proteome/metabolome analyses establish clinical relevance

**Figure 4:**
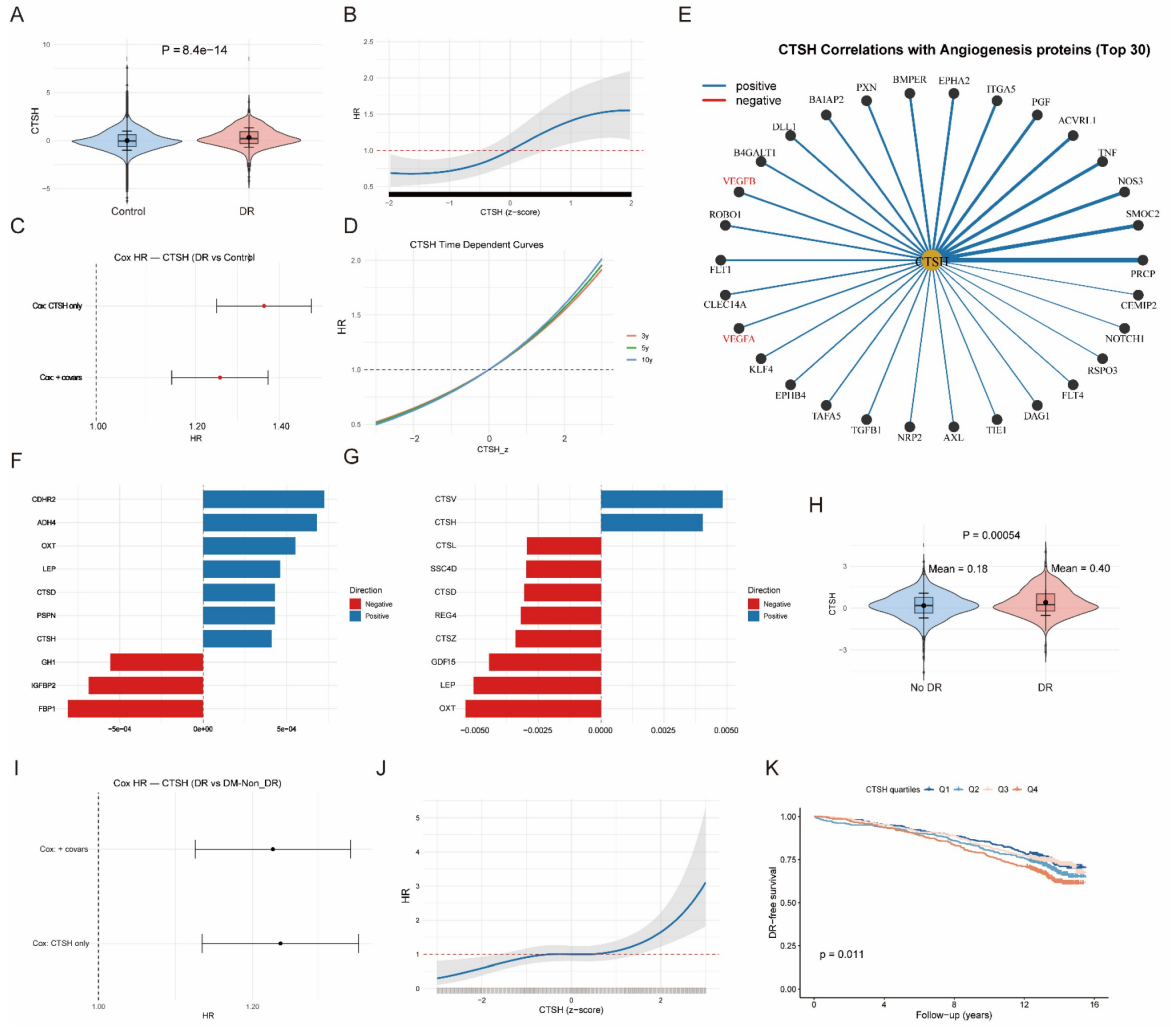
** Single-cell analyses reveal CTSH enrichment in myeloid compartments. (A)** UMAP projection of fibrovascular membrane single-cell transcriptomes highlighting immune and vascular cell clusters. **(B)** Dot plot showing CTSH expression across immune subsets. **(C)** Cell-type composition comparison between fibrovascular membranes and PBMC datasets. **(D-E)** Gene set enrichment analysis (GSEA) demonstrating enrichment of angiogenesis and inflammatory pathways in CTSH-high states. **(F-H)** Cross-cohort validation of CTSH expression across datasets and cell types. **(I)** Immune-cell-specific colocalization supporting CTSH regulatory variants in myeloid populations.

Across the UKB proteomics dataset, CTSH levels were right-shifted in DR compared with controls (Figure S4A), indicating globally higher abundance in cases. Mean ± SD standardized CTSH was 0.312 ± 1.016 in DR versus -0.005 ± 0.998 in controls (P = 8.6×10⁻¹⁴; Figure 4B; Table S13). In time-to-event analyses, CTSH independently predicted incident DR. Cox models yielded HR = 1.361 (95% CI 1.248-1.485) unadjusted and HR = 1.256 (95% CI 1.149-1.372) after multivariable adjustment. Logistic regression showed concordant effect sizes (OR = 1.364 and 1.259, respectively; Figure 4A; Table S14). These associations remained significant after adjustment for available demographic, lifestyle, cardiometabolic, and medication-related covariates, although residual confounding related to diabetes duration and treatment intensity cannot be fully excluded. When modeled continuously, CTSH demonstrated a monotonic dose-response relationship, with stronger associations over longer follow-up horizons (10 y > 5 y > 3 y) (Figure 4C-D). Proteome-wide correlation mapping positioned CTSH within angiogenic and immune-inflammatory networks (Tables S15 and S18), showing positive correlations with VEGF-family proteins and vascular mediators (ACVRL1, FLT1/KDR, NOS3, NOTCH, TEK), as well as inflammatory regulators (Figure 4E; Figure S4C-D). Proteome-metabolome integration further revealed associations with hyperglycemic and dyslipidemic signatures, including positive correlations with glycoprotein acetylation, glycolysis-related markers, and triglyceride/LDL measures, and inverse correlations with HDL composition, consistent with a systemic inflammatory-metabolic milieu. CTSH ranked among the top contributors distinguishing DR from controls in multivariate models (Figure 4F-G; Table S19).

Among individuals with diabetes only, CTSH remained elevated in DR compared with DM-NonDR (0.403 ± 0.927 vs 0.177 ± 0.890; P = 5.4×10⁻⁴; Figure 4J; Table S16). CTSH continued to predict incident DR in Cox models (HR = 1.240 unadjusted; HR = 1.237 adjusted) and logistic regression (OR = 1.291 and 1.287, respectively; Figure 4I; Table S17). A continuous dose-response persisted (Figure 4K), and baseline CTSH quartiles stratified DR-free survival, with Q4 exhibiting poorer prognosis (P = 0.011; Figure 4L). CTSH also remained a prominent driver of metabolic differentiation between DR and DM-NonDR, with canonical correlation analyses confirming coordinated proteome-metabolome shifts that were stronger in DR (Figure 4H; Tables S20-S22; Figures S4E-F).

### 3.7 CTSH regulates VEGF and oxidative stress under high glucose

**Figure 5:**
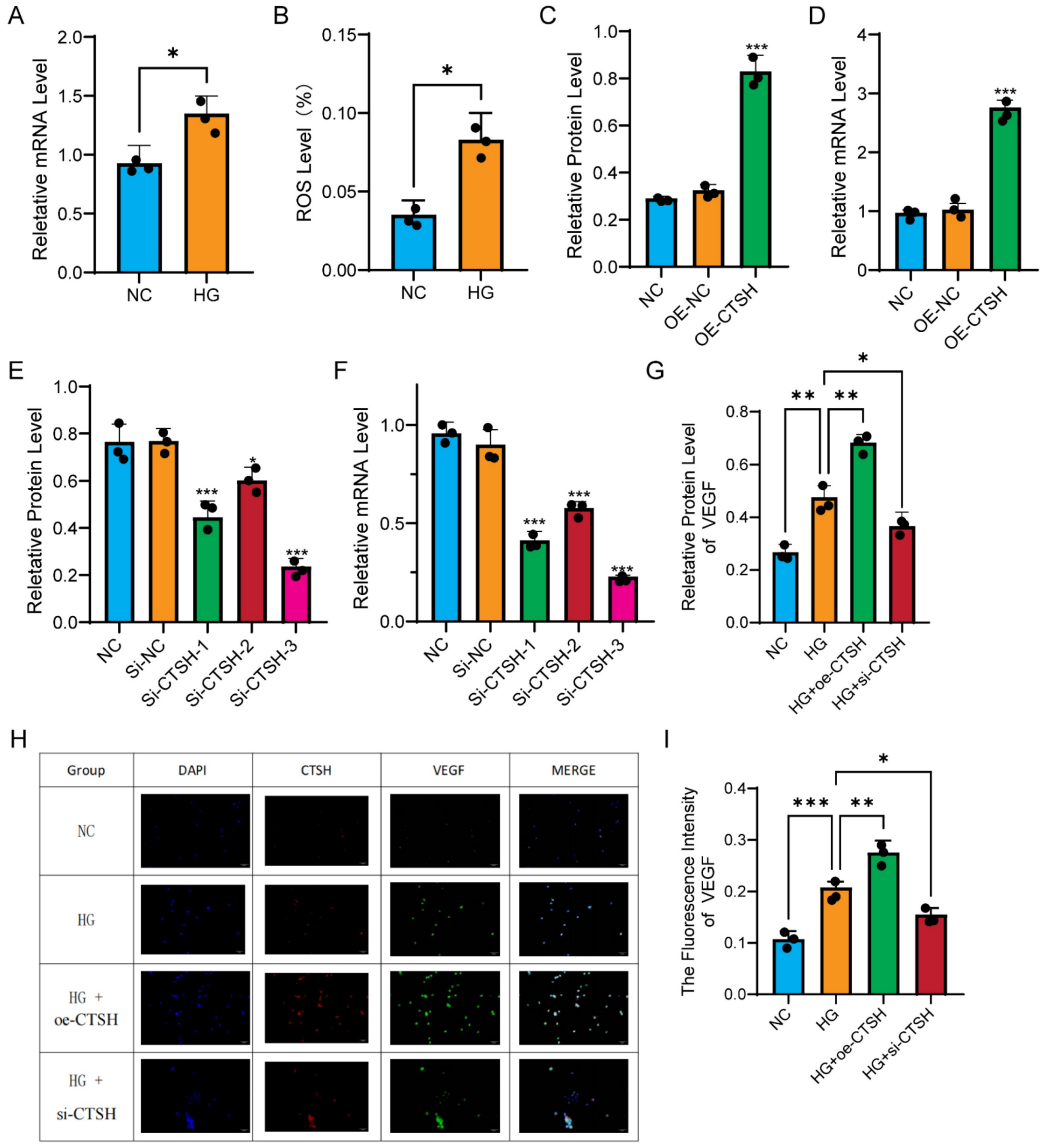
** High glucose induces CTSH-dependent oxidative and angiogenic responses in monocytes. (A-B)** High glucose (HG) increases CTSH expression and ROS levels compared to normal control (NC). **(C-D)** CTSH overexpression enhances VEGF expression at protein and transcript levels. **(E-F)** siRNA-mediated CTSH knockdown reduces CTSH protein and mRNA levels. **(G)** VEGF protein changes under HG with CTSH overexpression or knockdown. **(H-I)** Immunofluorescence staining and quantification of VEGF under experimental conditions.

**Figure 6:**
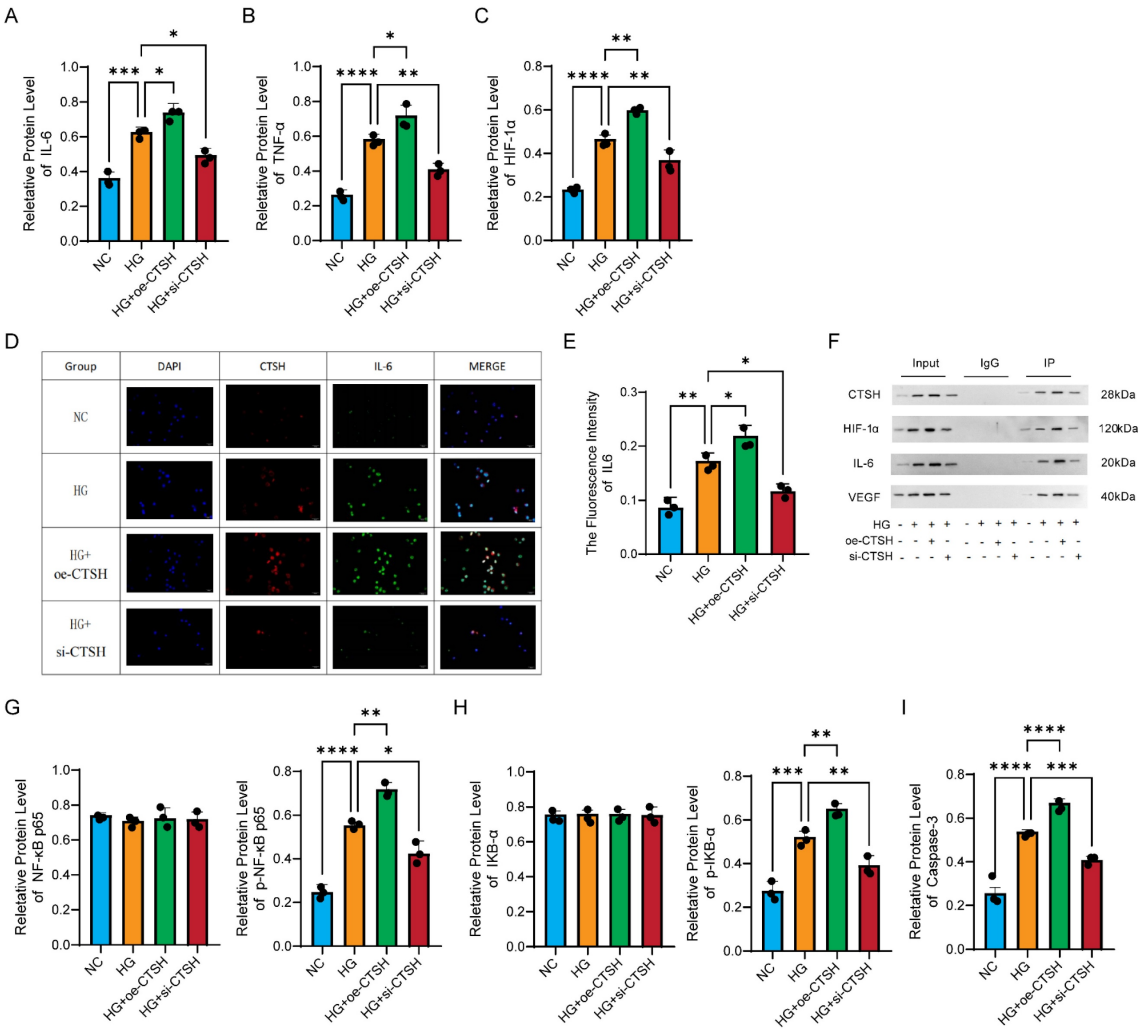
** CTSH regulates inflammatory, hypoxic, and NF-κB signaling pathways. (A-C)** Effects of CTSH modulation on IL-6, TNF-α, and HIF-1α protein expression under HG conditions. **(D-F)** Immunofluorescence and immunoprecipitation analyses supporting CTSH involvement in VEGF-associated signaling. **(G-I)** NF-κB pathway activity (p65, p-p65, IκBα, p-IκBα) following CTSH overexpression or silencing.

To further explore the functional relevance of CTSH upregulation in myeloid cells under diabetic conditions, we examined its expression and downstream effects in human monocyte THP-1 cells exposed to high glucose. In monocytes, high glucose significantly increased CTSH expression and ROS production. qPCR revealed that *CTSH* mRNA levels were elevated in HG compared with NC (Figure 5A), and ROS levels increased in parallel (Figure 5B). Overexpression of CTSH induced a further increase in CTSH protein (Figure 5C) and mRNA (Figure 5D), while three independent siRNA constructs efficiently suppressed CTSH at both protein (Figure 5E) and transcript levels (Figure 5F). VEGF expression closely followed CTSH modulation. Western blot demonstrated that VEGF protein levels increased under HG, were further enhanced with CTSH overexpression, and were significantly reduced by CTSH knockdown (Figure 5G). Immunofluorescence staining confirmed strong spatial co-localization of CTSH and VEGF under HG and HG plus overexpression, with signal attenuation in the CTSH knockdown group (Figure 5H). Quantification of VEGF fluorescence intensity verified these changes (Figure 5I). In addition, co-immunoprecipitation demonstrated that CTSH physically associated with VEGF (Figure 6F), supporting direct molecular interaction. Collectively, these data indicate that high glucose drives CTSH upregulation and oxidative stress, and that CTSH is required for robust VEGF induction and localization under diabetic conditions.

### 3.8 CTSH modulates inflammatory mediators

Inflammatory mediators exhibited the same regulation pattern as VEGF. Western blot analysis showed that IL-6 levels increased under HG, were further enhanced with CTSH overexpression, and declined after CTSH knockdown (Figure 6A). TNF-α followed the same trend (Figure 6B), and HIF-1α showed the highest expression in the HG plus overexpression group with partial reversal upon knockdown (Figure 6C). Immunofluorescence revealed stronger IL-6 signals in HG and HG with overexpression, which were reduced by CTSH knockdown (Figure 6D), with quantification confirming these differences (Figure 6E). Co-immunoprecipitation assays identified CTSH complexes containing IL-6 and HIF-1α as well as VEGF, supporting biochemical interactions with angiogenic and inflammatory mediators (Figure 6F).

Total NF-κB p65 and total IκB-α were relatively stable across groups, as shown in the left panels of Figures 6G and 6H. In contrast, the phosphorylated forms p-NF-κB p65 and p-IκB-α mirrored the cytokine and VEGF patterns, rising under HG and further with CTSH overexpression, then decreasing after CTSH knockdown (right panels of Figures 6G and 6H). Caspase-3 also followed this trend, with maximal activation in the HG plus overexpression group and reduction after CTSH knockdown (Figure 6I). Together, these results show that CTSH is not only required for the induction of inflammatory mediators but also promotes NF-κB signaling activation through phosphorylation events and Caspase-3 cleavage, without altering total protein abundance.

### 3.9 Structure-based screening identifies CTSH-binding small molecules that form stable complexes

**Figure 7:**
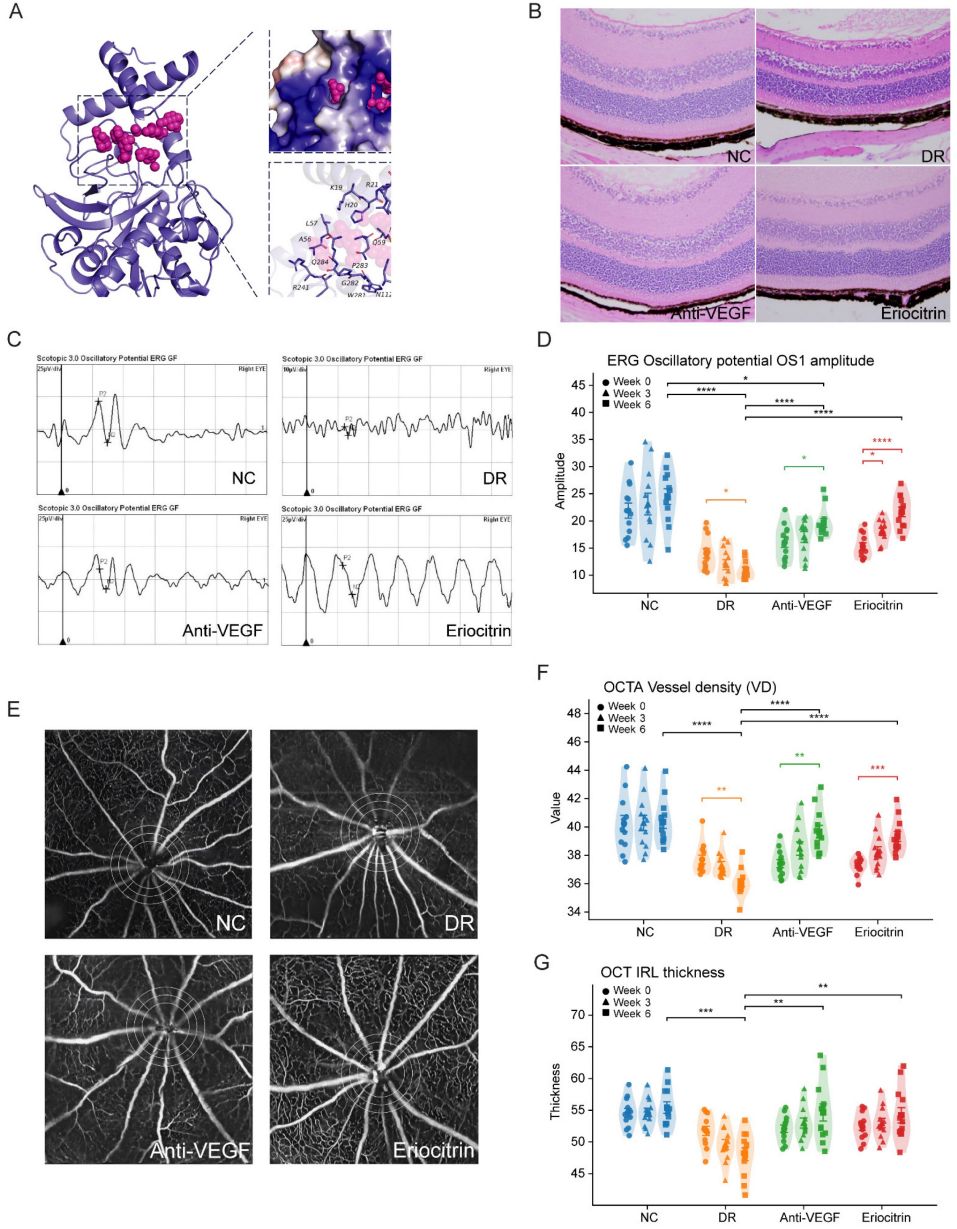
** CTSH-targeting candidate Eriocitrin improves retinal function, vascular integrity, and structure in db/db diabetic mice. (A)** Predicted molecular docking pose of Eriocitrin within the CTSH catalytic pocket, illustrating the binding conformation and key interacting residues identified by structure-based virtual screening. **(B)** Representative retinal hematoxylin-eosin (H&E) staining showing retinal laminar structure in normal control (NC), diabetic retinopathy (DR), anti-VEGF (Aflibercept), and Eriocitrin-treated groups. **(C)** Representative electroretinography (ERG) oscillatory potential (OP) waveforms from each experimental group. **(D)** Quantification of ERG oscillatory potential OS1 amplitudes measured longitudinally at baseline (week 0), week 3, and week 6 after intravitreal treatment. **(E)** Representative OCTA images illustrating retinal vascular networks across the four experimental groups. **(F)** Quantification of retinal vessel density (VD) derived from OCTA analyses at the indicated time points. **(G)** Optical coherence tomography (OCT) measurements of inner retinal layer thickness across groups and time points. Data are presented as mean ± SD. Statistical significance: *P < 0.05, **P < 0.01, ***P < 0.001.

Virtual screening of 24,123 compounds against CTSH identified a primary binding pocket comprising Lys19, Arg21, Lys22, Leu57, Ala56, Gln284, Arg241, Tyr240, Trp281 and surrounding residues. After filtering, 42 compounds were retained and top candidates were prioritized based on docking score and binding characteristics (Table S29). Two-dimensional interaction maps indicated stable hydrogen bonding and hydrophobic contacts with key pocket residues across top-ranked ligands (Fig. S6A-F). A representative docking pose illustrating occupancy of the CTSH pocket by a lead ligand is shown in Fig. 7A. Binding free energy calculations further supported favorable complex stability across candidates (Table S33), reinforcing CTSH as a tractable drug target for downstream functional evaluation. These results represent computational predictions of CTSH-ligand binding and do not constitute direct biochemical evidence of enzymatic inhibition.

### 3.10 MD-selected CTSH-binding candidates Suppress Inflammatory and Hypoxic Signaling in THP-1 Monocytes

Because biochemical inhibition assays were not performed, these compounds are interpreted as CTSH-targeting candidates rather than confirmed enzymatic inhibitors. We measured the expression of key inflammatory, hypoxic and apoptotic proteins in high-glucose-stimulated THP-1 monocytes following treatment with Eriocitrin, Tenuifoliside B or AP-III-a4 (Fig. S8). Compared to the normal glucose (NC) group, high glucose (HG) markedly increased caspase-3, HIF-1α, phosphorylated IkappaBα (p-IkappaBα), phosphorylated NF-kB p65 (p-p65), IL-6, and the p-IkappaBα/IkappaBα ratio, while total IkappaBα and NF-kB p65 levels remained relatively stable. Among the three compounds, Eriocitrin consistently showed the most substantial suppressive effect, reducing inflammatory and hypoxic signaling readouts toward baseline and indicating more potent inhibition of canonical NF-kB activation. Based on its consistent multi-pathway suppression *in vitro*, Eriocitrin was nominated for *in vivo* evaluation.

### 3.11 Intravitreal Eriocitrin achieves broad functional and vascular rescue in diabetic retinopathy *in vivo*

We next evaluated Eriocitrin *in vivo* using the db/db diabetic mouse model of diabetic retinal injury, initiating treatment at 12 weeks of age. At this stage, the model predominantly reflects early microvascular, inflammatory, and neuroretinal abnormalities rather than fully developed proliferative diabetic retinopathy. Compounds were administered by unilateral intravitreal injection into the vitreous cavity, and mice received either the comparator anti-VEGF control (aflibercept) or Eriocitrin followed by longitudinal ERG/OCTA/OCT profiling (Fig. 7). Histological examination using hematoxylin-eosin staining revealed diabetes-associated retinal structural alterations in untreated db/db mice, whereas both anti-VEGF and Eriocitrin treatment partially preserved retinal laminar organization and overall retinal integrity (Fig. 7B).

ERG analysis revealed that Eriocitrin elicited a robust improvement in inner-retinal signaling, with greater recovery of oscillatory potential OS1 amplitude compared with anti-VEGF across follow-up timepoints (Fig. 7C-D). Eriocitrin also improved additional functional endpoints, including scotopic b-wave (3.0) and 30-Hz flicker responses (Fig. S9A-B), consistent with restoration of retinal circuit performance.

In parallel, OCTA metrics demonstrated diabetes-associated vascular impairment that was significantly restored by Eriocitrin, including improved vessel density and complementary network features (Fig. 7E-F; Fig. S9C-E). OCT-based structural assessment further supported partial restoration of inner retinal layer features in treated animals (Fig. 7G; Fig. S9F). Together, these data indicate that intravitreal Eriocitrin delivers multi-dimensional rescue *in vivo*, matching anti-VEGF across several vascular and structural readouts while providing enhanced recovery of OS1 amplitude.

### 3.12 Eriocitrin suppresses high-glucose induced inflammatory, hypoxic and pro-angiogenic programs *in vivo*

**Figure 8:**
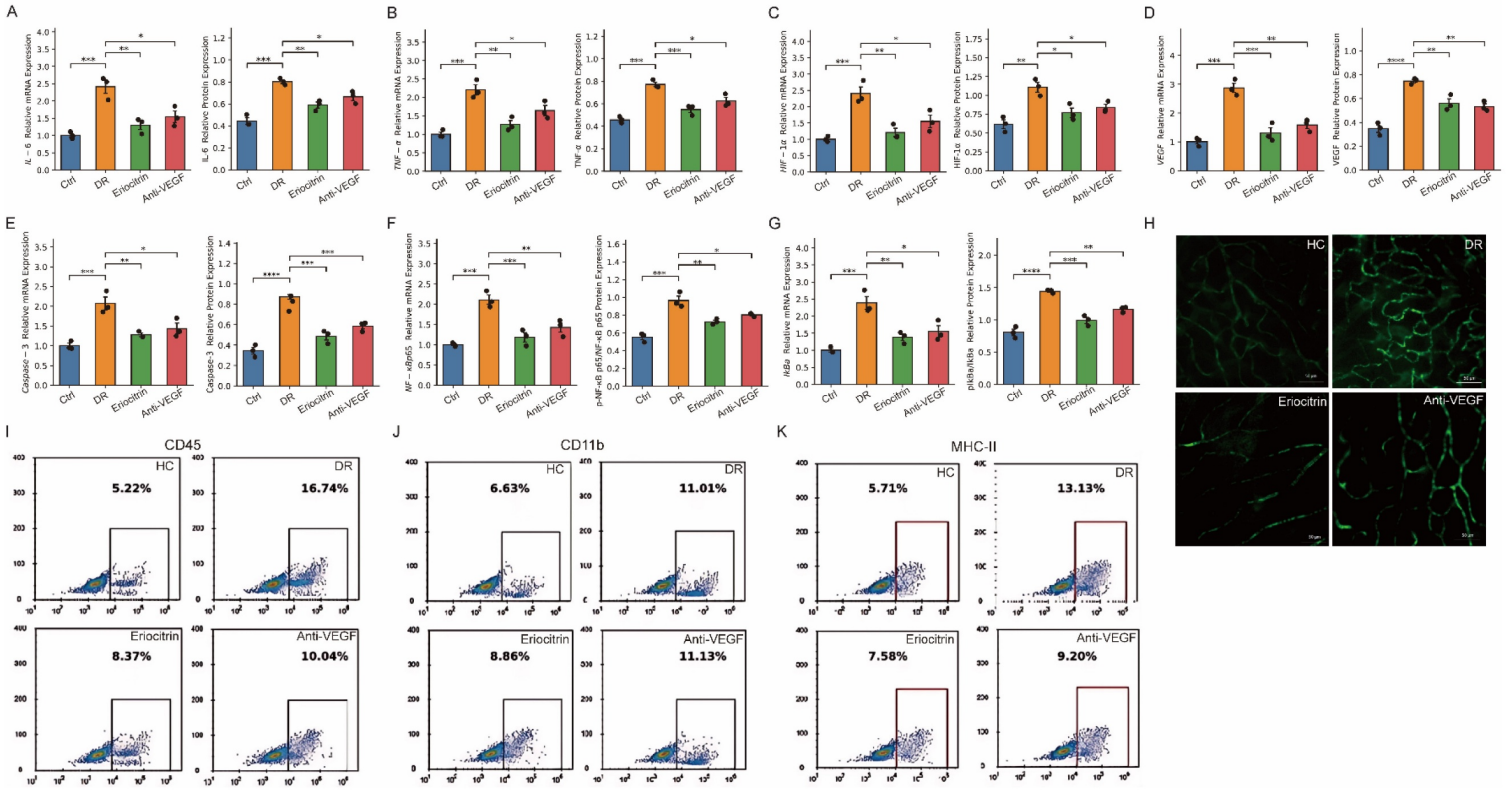
** Eriocitrin attenuates inflammatory, hypoxic, and NF-κB-associated signaling and reduces immune cell infiltration in diabetic retina. (A-B)** mRNA and protein expression levels of the inflammatory cytokine IL-6 in retinal tissue across experimental groups. **(C-D)** mRNA and protein expression levels of TNF-α. **(E)** mRNA and protein expression levels of Caspase-3, indicating modulation of apoptosis-related signaling. **(F)** mRNA and protein levels of NF-κB p65 and the phosphorylation ratio p-NF-κB p65/NF-κB p65. **(G)** mRNA and protein levels of IκBα and the phosphorylation ratio p-IκBα/IκBα. **(H)** Representative retinal vascular leakage imaging demonstrating vascular permeability in normal control (HC), diabetic retinopathy (DR), Eriocitrin-treated, and anti-VEGF groups. **(I-K)** Flow cytometry analyses showing immune cell infiltration in retinal tissue, including CD45⁺ leukocytes, CD11b⁺ myeloid cells, and MHC-II⁺ activated immune cells. Data are presented as mean ± SEM with individual points representing biological replicates. Statistical comparisons were performed using one-way ANOVA with post-hoc testing. *P < 0.05, **P < 0.01, ***P < 0.001.

To connect phenotypic rescue to pathway modulation, we profiled inflammatory, hypoxic/angiogenic and NF-κB signaling pathways *in vivo*. Diabetic retinal tissues showed marked up-regulation of IL-6 and TNF-α, accompanied by increased HIF-1α and VEGF, consistent with a coupled inflammatory-hypoxic pro-angiogenic state (Fig. 8A-D). Eriocitrin significantly reduced IL-6 and TNF-α at both transcript and protein levels and concurrently suppressed HIF-1α and VEGF expression, indicating attenuation of upstream inflammatory and hypoxic signaling. At the signaling level, caspase-3 expression was also reduced in the Eriocitrin-treated group (Fig. 8E). Eriocitrin further diminished canonical NF-κB activation signatures, including reduced NF-κB p65 abundance and a decreased p-NF-κB p65/NF-κB p65 ratio, together with suppression of IκBα phosphorylation dynamics (p-IκBα/IκBα) (Fig. 8F-G).

Consistent with these molecular changes, vascular permeability assays revealed marked glucose leakage in diabetic retinas that was substantially reduced by Eriocitrin treatment (Fig. 8H), indicating improved vascular barrier integrity. Flow cytometry further demonstrated increased immune cell infiltration in diabetic retinas, including elevated CD45⁺ leukocytes, CD11b⁺ myeloid cells and MHC-II⁺ activated immune cells, which were significantly attenuated following Eriocitrin treatment (Fig. 8I-K). Together, these findings support a model in which Eriocitrin suppresses diabetes-induced inflammatory and immune activation and acts upstream of pathological neovascular signaling by dampening the NF-κB-HIF-1α-VEGF axis, thereby restoring vascular integrity and limiting inflammatory cell infiltration in diabetic retinopathy.

## Discussion

Our study demonstrates that CTSH upregulation in myeloid cells triggers potent inflammatory signaling in the diabetic retina. Under high-glucose conditions, we observed increased CTSH expression in THP-1 monocytes, accompanied by NF-κB pathway activation and elevated secretion of pro-inflammatory cytokines (IL-6 and TNF-α). This aligns with NF-κB's known role as a central transcriptional hub for inflammatory mediators in PDR 64. By activating NF-κB, CTSH establishes an autocrine and paracrine loop in which monocyte-derived IL-6 and TNF-α further amplify inflammatory cell recruitment and cytokine release. In diabetic retinal tissues, these cytokines have been implicated in the chronic inflammation that characterizes PDR progression 65. Notably, innate immune cells in the diabetic retina not only secrete cytokines but also release proteases, exacerbating tissue injury. Consistent with this, microglia and macrophages in PDR secrete proteolytic enzymes and reactive oxygen species (ROS) alongside cytokines 66. Our findings suggest that CTSH may function as an upstream regulator of inflammatory signaling across DR, with genetic analyses providing the strongest support for its relevance to advanced proliferative disease. Overexpression of CTSH in monocytes enhanced IL-6 and TNF-α output, whereas silencing CTSH attenuated these responses, supporting a potential role for CTSH in promoting inflammatory activation in the diabetic retina.

Our data further suggest that CTSH-associated inflammatory signaling may contribute to blood-retinal barrier dysfunction 67. In both cellular and *in vivo* experiments, CTSH upregulation was accompanied by increased IL-6, TNF-α, and NF-κB activation, whereas Eriocitrin treatment reduced inflammatory signaling and vascular leakage 68-70. These findings support a model in which CTSH acts upstream of cytokine-mediated vascular destabilization and immune cell recruitment. Rather than indicating a single direct mechanism, our results place CTSH within a broader myeloid inflammatory program likely to exacerbate endothelial dysfunction, barrier breakdown, and retinal injury in diabetes. We also found that CTSH upregulation under high-glucose conditions was accompanied by increased ROS production, linking CTSH to oxidative stress in diabetic retinal injury. Together with the observed activation of NF-κB and induction of inflammatory cytokines, these data support a feed-forward model in which CTSH amplifies interconnected oxidative and inflammatory responses 71. This is consistent with the concept that diabetic retinal damage is sustained not by a single pathway, but by mutually reinforcing stress programs that couple redox imbalance, innate immune activation, and vascular dysfunction.

A key finding of the present study is that CTSH links myeloid activation to hypoxic and angiogenic signaling. In THP-1 cells, CTSH modulation altered HIF-1α and VEGF expression, while single-cell analyses localized CTSH predominantly to myeloid compartments within fibrovascular membranes enriched for inflammatory and angiogenic programs. *In vivo*, Eriocitrin suppressed HIF-1α/VEGF-related signaling together with inflammatory readouts. Taken together, these results support CTSH as an upstream regulator positioned at the interface of myeloid inflammation, hypoxic adaptation, and pro-angiogenic signaling, rather than as a downstream marker of VEGF activation alone. Overall, our findings support a convergent disease model in which CTSH sits upstream of interconnected inflammatory, oxidative, hypoxic, and angiogenic pathways. This integrative interpretation is supported by the consistency across genetic prioritization, UKB proteomics, single-cell localization, cell-based perturbation assays, and *in vivo* pharmacologic intervention. The translational implication is that CTSH may represent a multi-pathway target capable of modulating disease-driving biology beyond VEGF alone.

Finally, our study provides *in vivo* proof-of-concept that targeting CTSH may interrupt the inflammation-hypoxia-angiogenesis cycle in diabetic retinopathy. A single intravitreal administration of Eriocitrin in db/db mice improved retinal function and microvasculature across multiple readouts, including enhanced recovery of ERG oscillatory potential OS1 amplitude and restoration of OCTA-derived vascular metrics. Terminal retinal profiling further demonstrated coordinated suppression of inflammatory cytokines (IL-6, TNF-α), hypoxic/angiogenic mediators (HIF-1α, VEGF), apoptotic signaling (caspase-3), and canonical NF-κB activation signatures, supporting an upstream multi-pathway mechanism distinct from VEGF blockade alone. Clinically, however, anti-VEGF therapy for proliferative diabetic retinopathy is typically administered as repeated injections rather than as a single dose. Therefore, the present head-to-head comparison should be interpreted cautiously, as our single-injection design does not fully recapitulate real-world anti-VEGF treatment paradigms and may underestimate the clinical efficacy of sustained VEGF suppression. Within this experimental setting, Eriocitrin showed efficacy comparable to aflibercept across several vascular and structural endpoints and greater recovery of OS1 amplitude, but these findings should be regarded as proof-of-concept rather than definitive therapeutic superiority. Further studies are needed to define ocular target engagement, pharmacokinetics, durability, safety, and the potential benefit of CTSH inhibition alone or in combination with repeated anti-VEGF therapy.

### Study limitations

Several limitations should be considered. First, although high-glucose experiments consistently supported CTSH-associated inflammatory, hypoxic, and angiogenic signaling in THP-1 monocytes, osmotic controls such as mannitol or L-glucose were not included. Therefore, a potential contribution of osmotic stress cannot be fully excluded. In addition, THP-1 cells represent a simplified monocyte-lineage model and do not fully recapitulate the multicellular retinal microenvironment, which includes retinal endothelial cells, Müller glia, resident microglia, and other immune and stromal populations. Accordingly, the cellular findings should be interpreted as evidence for myeloid-associated CTSH biology rather than a complete model of retinal pathophysiology. Second, although co-immunoprecipitation experiments suggested interactions between CTSH and VEGF, IL-6, and HIF-1α, further biochemical validation is required. Reciprocal Co-IP and direct testing of whether CTSH inhibition reduces complex formation and downstream signaling were not performed. Third, the drug-discovery component should be interpreted cautiously. Virtual screening and molecular dynamics analyses identify candidate CTSH-binding compounds but do not provide direct biochemical evidence of enzymatic inhibition. Likewise, cellular assays demonstrating pathway suppression do not establish these compounds as confirmed CTSH inhibitors. Biochemical potency (e.g., IC50) and selectivity against other cathepsins were not evaluated in the present study and will require further investigation. Fourth, in the UKB analyses, although multivariable models were used, some clinically relevant diabetes-related covariates—such as diabetes duration, insulin therapy, lipid-lowering treatment, and antihypertensive treatment—were not uniformly available or incorporated in all models; therefore, residual confounding cannot be excluded. Finally, intravitreal validation was performed in db/db mice using a single-dose paradigm with relatively short follow-up. Moreover, the db/db model at 12 weeks of age primarily reflects early diabetic retinal abnormalities rather than fully developed proliferative disease. Therefore, the *in vivo* findings are most appropriately interpreted as evidence for effects on early diabetic retinal dysfunction and inflammation rather than as direct reversal of established PDR. Future studies should further evaluate dose-response relationships, repeated-dosing regimens, pharmacokinetics, target engagement, long-term safety, and efficacy in models that better recapitulate advanced proliferative retinopathy.

## Conclusion

This study identifies CTSH as a genetically supported and functionally validated upstream regulator of DR-related inflammatory, oxidative, and angiogenic signaling. Genetic analyses provided the strongest evidence for a role of CTSH in PDR, whereas UKB, cellular, and *in vivo* experiments supported broader relevance to DR-associated disease biology. CTSH integrates oxidative stress, inflammation, and angiogenic signalling through NF-κB and the HIF-1α/VEGF axis within myeloid compartments, forming a self-reinforcing pathogenic circuit. Leveraging structure-guided screening, we nominated Eriocitrin as a lead CTSH-targeting candidate and demonstrate that intravitreal Eriocitrin provides multi-dimensional rescue in db/db mice—improving retinal function and microvascular metrics while suppressing inflammatory, hypoxic/angiogenic, and NF-κB signatures in the retina. Collectively, these findings support CTSH targeting as an upstream, multi-pathway therapeutic strategy that may be relevant across diabetic retinopathy, with particular translational interest for advanced proliferative disease.

## Supplementary Material

Supplementary figures.

Supplementary tables.

## Figures and Tables

**Figure 1:**
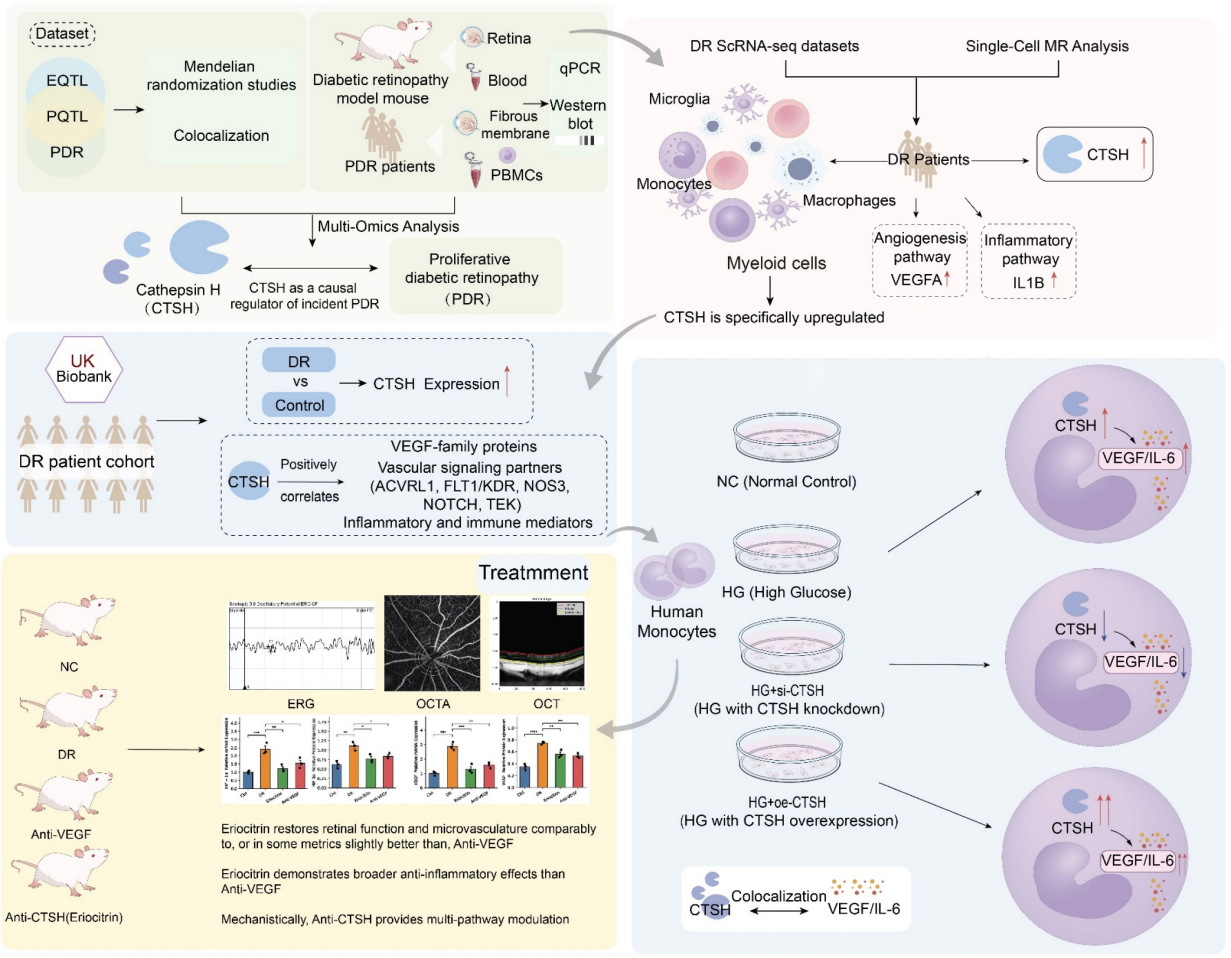
** Multi-omics framework identifies CTSH as a causal regulator of proliferative diabetic retinopathy (PDR). (A)** Study design integrating GWAS, eQTL, pQTL, and PDR datasets through Mendelian randomization (MR) and colocalization analyses, followed by experimental validation in diabetic mouse models, human fibrovascular membranes, peripheral blood mononuclear cells (PBMCs), and in vitro systems. **(B)** Single-cell RNA-seq datasets from DR fibrovascular membranes showing myeloid-enriched CTSH expression and association with angiogenesis and inflammatory signaling pathways. **(C)** Conceptual framework illustrating CTSH as a causal regulator of incident PDR. **(D)** UK Biobank analysis comparing CTSH expression between DR and controls. **(E)** Correlation network showing positive associations between CTSH and VEGF-family proteins, vascular signaling mediators (ACVRL1, FLT1/KDR, NOS3, NOTCH, TEK), and inflammatory/immune regulators. **(F)** Proposed mechanistic model in which CTSH integrates inflammatory and hypoxic signaling upstream of VEGF, forming a feed-forward pathogenic loop.

**Table 1:** Integrated multi-omics evidence and tiering of candidate genes.

Gene	SMR (Finland)				SMR (8cohort)		eQTL MR		Colocalization	Category
	CIS-eQTL		Whole Blood eQTL		Replication (CIS-eQTL)		Finland	8cohort	PP4 > 0.5 (weak) PP4 > 0.8 (strong)	
	pSMR (FDR)	pHEIDI	pSMR (FDR)	pHEIDI	pSMR (FDR)	pHEIDI	pval	pval		
CTSH	1.88E-02 √	6.11E-01 √	1.82E-02 √	5.94E-02 √	9.66E-14 √	4.46E-01 √	9.04E-06 √	5.29E-03 √	Weak (0.67) √	Tier 1
CYP21A2	8.72E-16 √	2.55E-04 ×	1.74E-09 √	6.06E-02 √	1.73E-02 √	4.80E-01 √	6.40E-04 √	2.11E-02 √	No ×	Tier 2
MICB	4.68E-04 √	8.66E-02 √	4.67E-03 √	9.39E-02 √	3.13E-05 √	1.39E-01 √	No ×	No ×	No ×	Tier 2
CCNE2	3.34E-02 √	7.31E-01 √	No ×	No ×	9.91E-01 ×	5.48E-01 ×	3.48E-02 √	9.97E-01 ×	Strong (0.86) √	Tier 2
TP53INP1	1.22E-02 √	1.04E-03 ×	4.92E-02 √	7.88E-01 √	9.96E-01 ×	3.89E-01 ×	No ×	No ×	Strong (0.90) √	Tier 2
PPIP5K2	4.49E-02 √	4.00E-01 √	6.74E-02 ×	6.09E-01 ×	9.72E-01 ×	3.16E-01 ×	No ×	No ×	Weak (0.75) √	Tier 3
XRCC1	1.05E-02 √	9.65E-02 √	8.72E-01 ×	4.77E-02 ×	9.95E-01 ×	6.33E-01 ×	No ×	8.34E-01 ×	Strong (0.96) √	Tier 3
C4B	4.27E-18 √	5.08E-13 ×	1.75E-12 √	2.29E-02 ×	9.96E-01 ×	9.99E-01 ×	2.61E-06 √	No ×	No ×	Tier 3
C4A	7.70E-17 √	1.48E-07 ×	1.75E-12 √	2.73E-05 ×	9.87E-01 ×	3.66E-01 ×	7.57E-08 √	No ×	No ×	Tier 3
HLA-C	1.12E-06 √	1.19E-08 ×	1.97E-09 √	7.79E-03 ×	9.73E-01 ×	2.25E-01 ×	6.33E-03 √	4.37E-01 ×	No ×	Tier 3
ZBTB22	7.43E-03 √	3.01E-05 ×	4.44E-02 √	3.69E-01 √	No ×	No ×	No ×	No ×	No ×	Tier 3

Table 1. Integrated multi-omics evidence for candidate genes associated with proliferative diabetic retinopathy (PDR). Genes are classified into Tier 1 (CTSH, strongest evidence), Tier 2 (moderate evidence), and Tier 3 (limited evidence) according to fulfillment of discovery and validation criteria across SMR, two-sample MR, and colocalization analyses. The symbol “√” indicates that the corresponding criterion was met, whereas “×” indicates that the criterion was not met. PP4 > 0.8 indicates strong colocalization support, and PP4 > 0.5 indicates moderate support.

## Data Availability

All data generated or analyzed during this study are included in the published article and its supplementary tables. Detailed descriptions of the datasets and variables used are provided in the Methods section. No additional datasets were generated or analyzed beyond those reported.
