# Supplementary material for: Cathepsin H drives hypoxia-associated inflammatory and angiogenic programs in diabetic retinopathy and represents a potential therapeutic target
Source: Int J Biol Sci. 2026 Apr 23;22(9):4647–69. doi: 10.7150/ijbs.134125 (PMC13182238; doi:10.7150/ijbs.134125)
Supplement: Supplementary file 1 — Supplementary figures. [file ijbsv22p4647s1.pdf]

Supplemental Figures

Figure S1

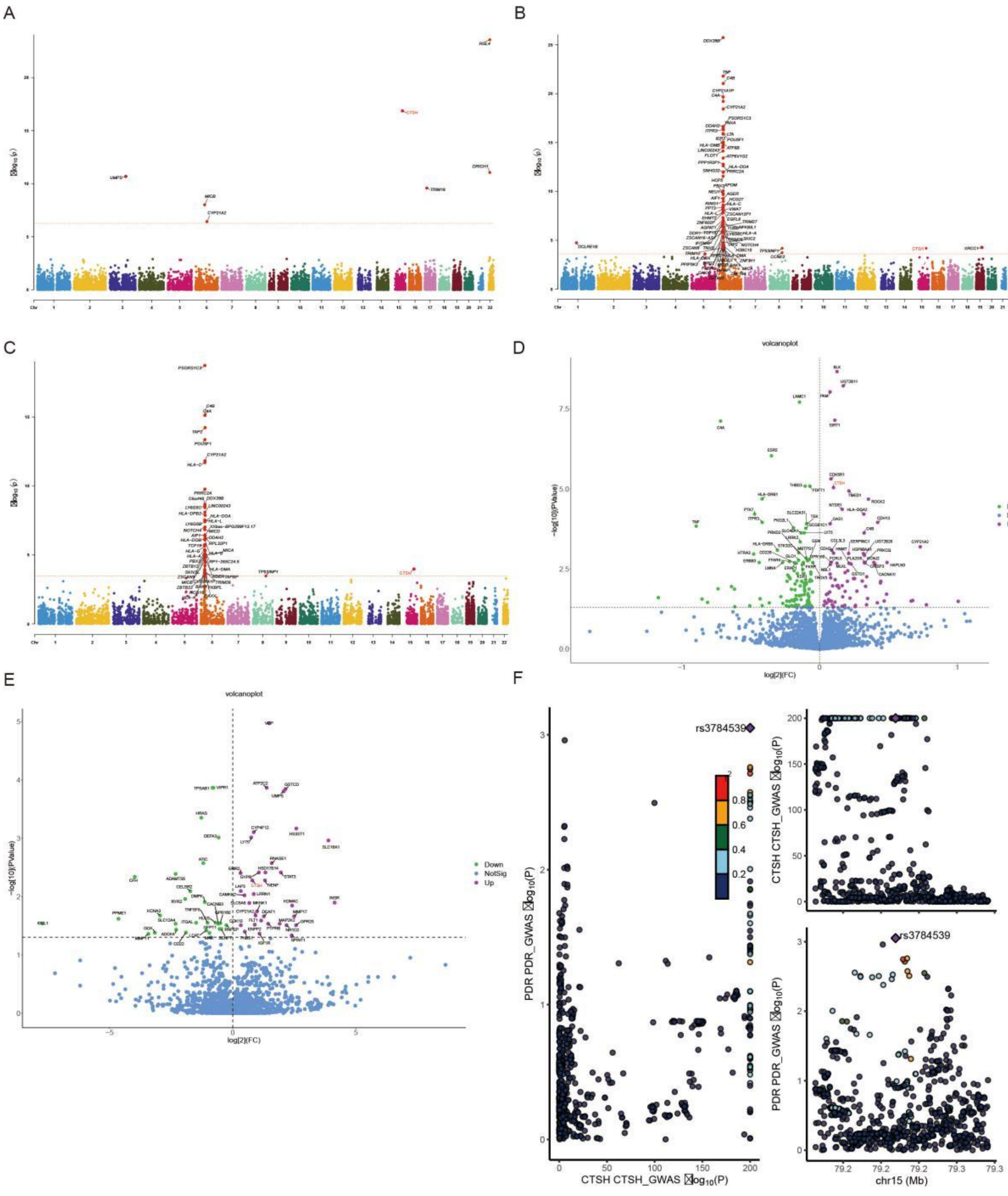

Figure S1. Genome-wide association and differential expression analyses

(A–C) Manhattan plots highlighting CTSH locus significance.

(D–E) Volcano plots showing differential gene expression in DR.

(F) Locus-specific visualization of CTSH-associated variants.

Figure S2

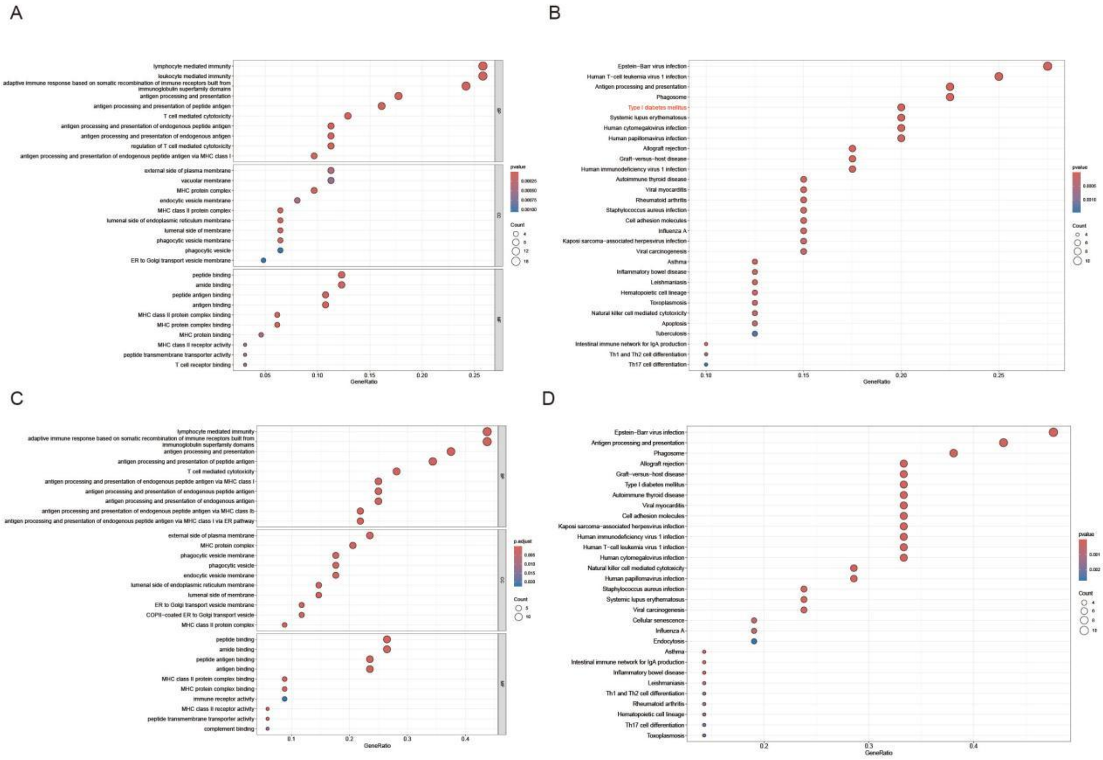

Figure S2. Functional enrichment analyses of CTSH-associated genes

(A–C) Gene ontology (GO) enrichment for immune and antigen presentation pathways.

(B–D) KEGG pathway enrichment highlighting inflammatory and autoimmune signaling.

Figure S3

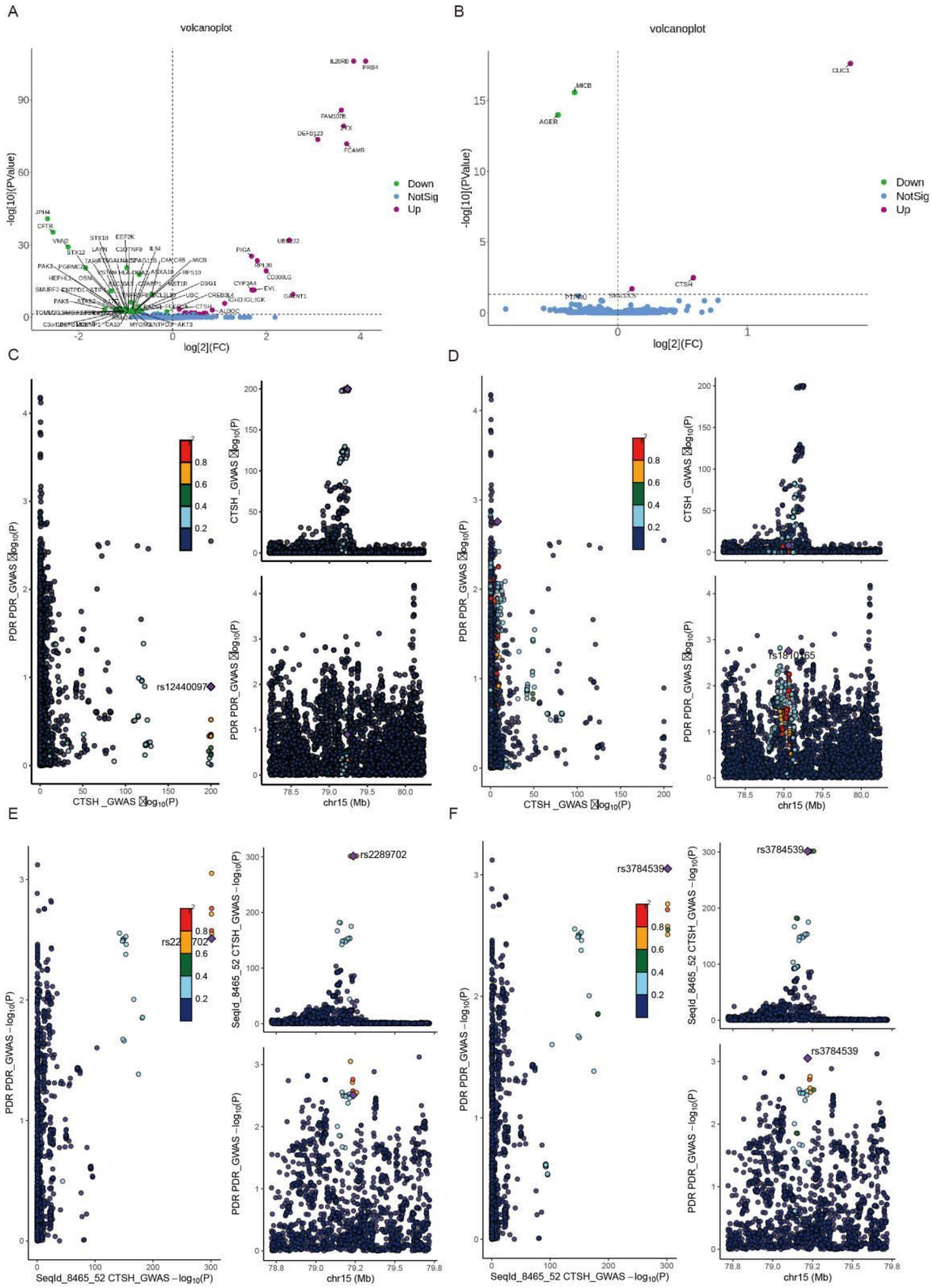

Figure S3. Additional locus and fine-mapping analyses

(A–B) Differential expression volcano plots.

(C–F) Regional association and fine-mapping plots supporting CTSH as a candidate causal locus.

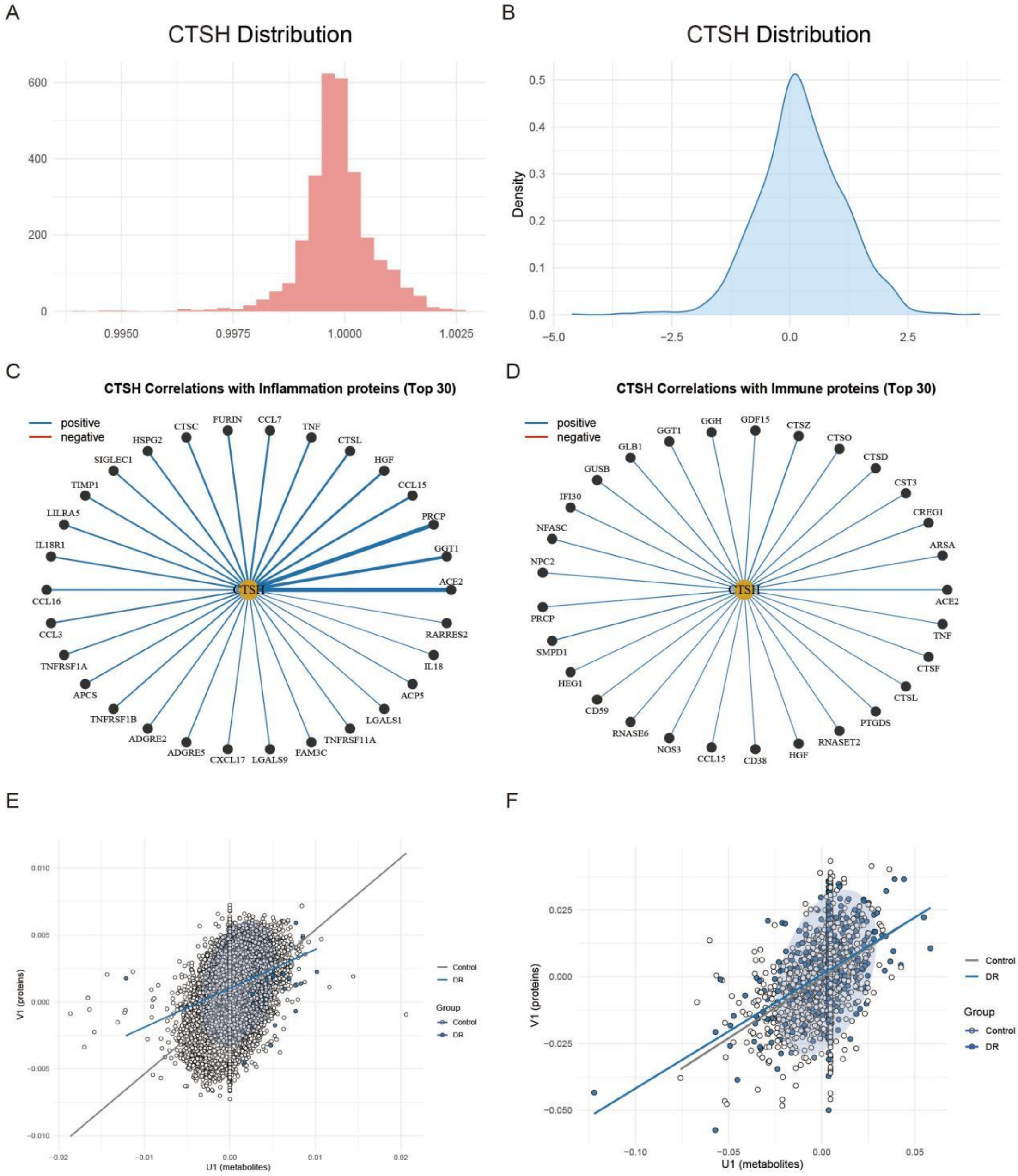

Figure S4. Distribution and correlation patterns of CTSH

(A–B) Distribution density of CTSH levels.

(C–D) Correlation networks between CTSH and inflammatory/immune proteins.

(E–F) Joint metabolite–protein association scatter plots.

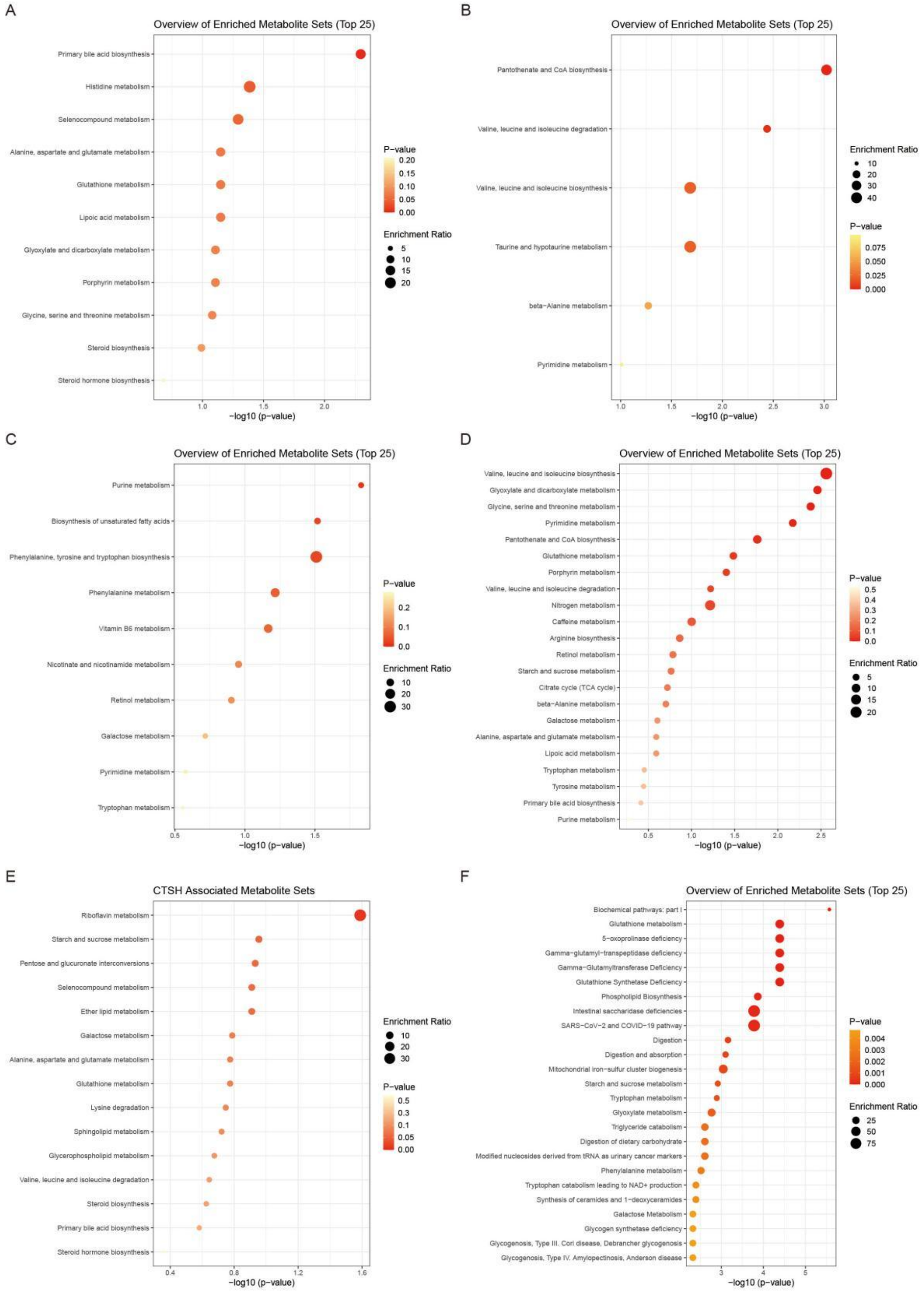

Figure S5. Metabolite enrichment analyses associated with CTSH

(A–D) Overview of enriched metabolite sets.

(E) CTSH-associated metabolic pathways.

(F) Expanded pathway enrichment overview.

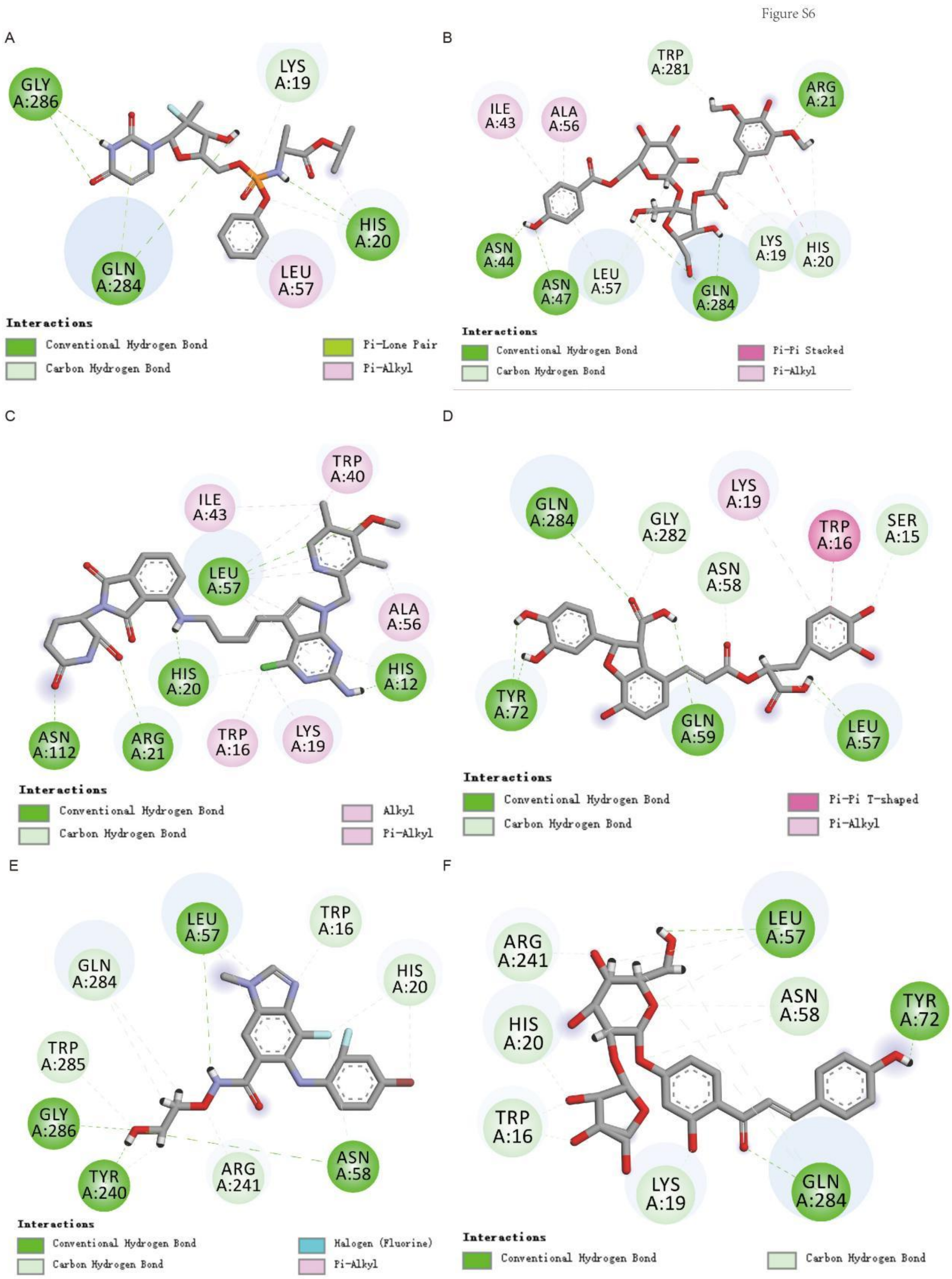

Figure S6. Molecular docking interaction maps

(A–F) Two-dimensional interaction maps of top-ranked CTSH-binding compounds showing hydrogen bonding and hydrophobic interactions.

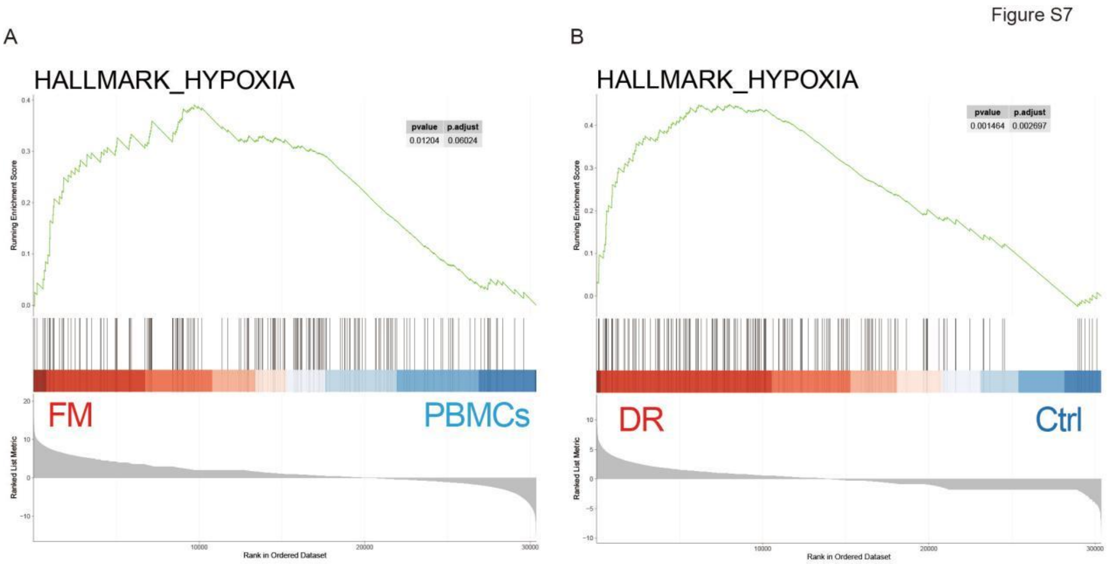

Figure S7. GSEA analysis of hypoxia signatures

(A–B) HALLMARK_HYPOXIA enrichment comparing fibrovascular membranes vs PBMCs and DR vs control.

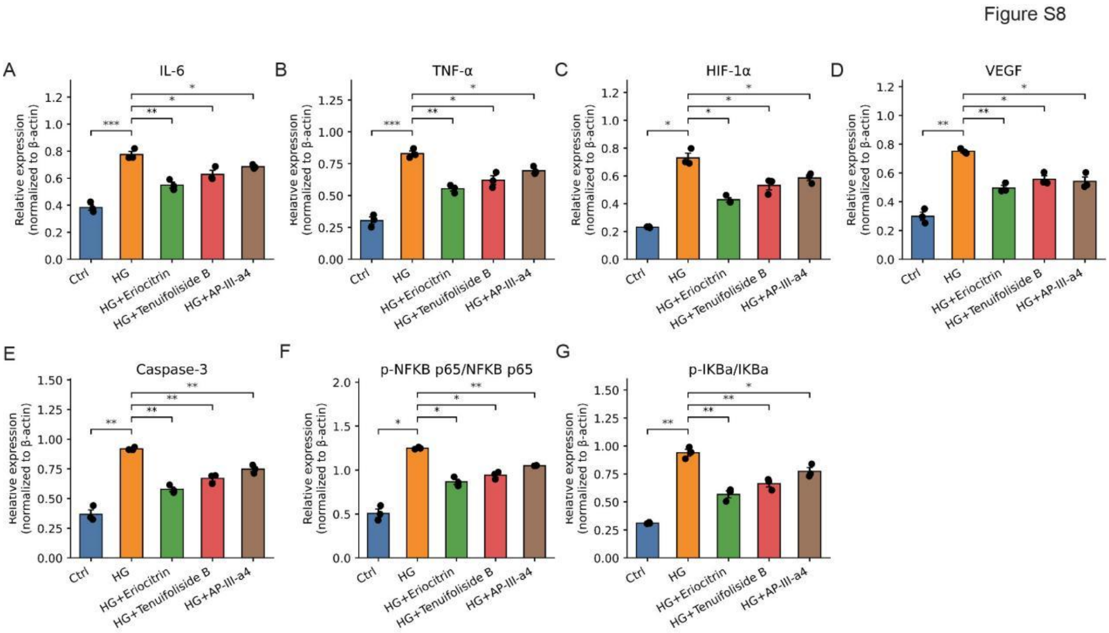

Figure S8. In vitro evaluation of CTSH-targeting compounds

(A–D) Effects of Eriocitrin, Tenuifoliside B, and AP-III-a4 on IL-6, TNF-α, HIF-1α, and VEGF expression under high glucose.

(E–G) Caspase-3, NF-κB p65, and p-IκBα/IκBα ratios.

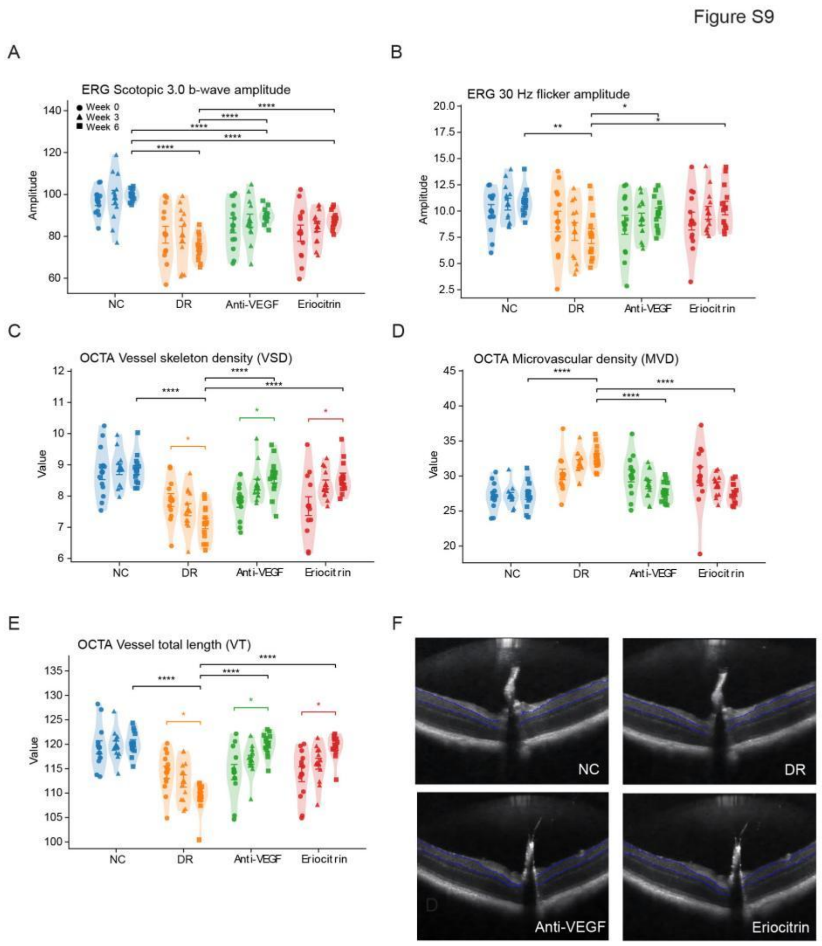

Figure S9. Additional in vivo functional and vascular measurements

(A) Scotopic 3.0 b-wave amplitude.

(B) 30-Hz flicker amplitude.

(C–E) Vessel skeleton density, microvascular density, and vessel total length.

(F) Representative OCT structural images.
